# Navigating the challenges of passenger name record data and the way forward

**DOI:** 10.12688/openreseurope.18037.1

**Published:** 2024-08-05

**Authors:** Christiana Aposkiti, Freideriki Makri

**Affiliations:** 1 P.Kanellopoulou Str.4, KEMEA, Center for Security Studies, Athens, 10177, Greece; 2 P.Kanellopoulou Str.4, Hellenic Police, Athens, 10177, Greece

**Keywords:** PIUS Challenges, Travel Intelligence Recommendations, Passenger Name Record (PNR), Passenger Information Units (PIUs), counterterrorism, crime prevention, EU PNR Directive, Advance Passenger Information (API)

## Abstract

**Background:**

The TENACITy EU-funded project investigates the multifaceted challenges surrounding the utilisation of Passenger Name Record (PNR) data following the implementation of the PNR Directive (EU) 2016/681, which marks a significant shift in EU air travel intelligence.

**Methods:**

This study employed a combination of research and survey methodologies to gather data from various stakeholders involved in the implementation of the PNR Directive. The survey focused on identifying the key obstacles faced by Passenger Information Units (PIUs), including the absence of standardised practices and issues of data quality.

**Results:**

The findings highlight two primary obstacles confronting PIUs: the lack of standardised practices among stakeholders and the poor quality of PNR data. Additionally, fragmented implementation and regulatory barriers were identified as significant hindrances to the optimal utilisation of PNR data for counterterrorism and crime prevention efforts.

**Conclusions:**

Addressing these challenges requires nuanced solutions, with technological tools presenting potential remedies to operational constraints. There is a collective call for mandating specific data elements to enhance the effectiveness of PNR data utilisation. This paper provides insights and recommendations to enhance PIUs' capabilities, contributing to the ongoing discourse on EU travel intelligence.

## Introduction

The 9/11 attack signified a new era on the field of travel intelligence which refers to ‘the systematic collection, analysis, and utilisation of travel-related information and intelligence to enhance security measures and law enforcement efforts’ ( Kanellopoulos, 2024) since it highlighted the importance of the processing of Passenger Name Record (PNR) data, which prior to the attack were mainly collected by the air carriers in a non-systematic way to book air travel reservations, to fight and even prevent terrorism and organised crime ( De Hert & Papakonstantinou, 2010). The attack led to the implementation of the Aviation and Transportation Security Act in 2001 by the United States ( US Government Publishing Office, 2001), which consequently opened the path of the air travel intelligence landscape as it is known today.

The European Union (EU) has undertaken efforts in border management embodying a multifaceted approach to safeguarding its borders and combating terrorism and serious cross border crime. More recently, these efforts include the Entry Exit System (EES) ^1^ and the European Travel Information and Authorisation System (ETIAS) which are not still in operation ^2^. Moreover, foundational to the EU’s border security architecture are the Advance Passenger Information (API) Directive ( The Council of the European Union, 2004), and the Schengen Borders Code ( The European Parliament & the Council of the European Union, 2016b), which aim at enhancing information and intelligence sharing with third countries concerning the movement of individuals across borders. In this context, the use of PNR data has been integral to the international cooperation efforts of the EU against terrorism and serious crime for nearly two decades with the implementation of the Passenger Name Records (PNR) Directive (EU) 2016/681 (henceforth “the Directive”) being the cornerstone of them. The Directive regulates the use of PNR data in the European Union for the prevention, detention, investigation and prosecution of terrorist offences and serious crime and calls for each Member State to establish a Passenger Information Unit (PIU) ( The European Parliament & the Council of the EU, 2016a). The PIU serves as the sole authority for collecting, storing, processing, and transferring under specific conditions PNR and API data ^3^ to other PIUs, National Competent Authorities (NCAs), Europol, and third countries. This process must fully respect fundamental rights, including privacy and personal data protection.

The European Commission's Review of the Directive ( European Commission, 2020b) highlights the increasing recognition globally, not limited to EU Member States, of the utility of PNR data as a tool for law enforcement in the fight against terrorism and serious crime. Yet, the findings also showed that the implementation of the Directive faces delays and fragmentation. Data quality and accuracy were considered paramount for improving the collection of relevant data used to combat terrorism and serious crime. Despite EU legislation, variations in perception and methodologies in implementing the Directive were reported. PIUs officers outlined incomplete, inaccurate, or untimely data entry, while the ‘broad’ and ‘relatively unclear formulation’ of the Directive was considered the main reason why PIUs did not move to spontaneous transfers ( European Commission, 2020a).

In this complex travel intelligence landscape, the Travel Intelligence Against Crime and Terrorism - TENACITy project ^4^ envisions to address key challenges by developing innovative tools, offering trainings, and establishing a holistic approach to prevent and fight terrorism and crime through travel intelligence. The present document reports on the findings of a survey conducted in the context of the TENACITy project, that was addressed to the PIUs, one of the main actors of today’s travel intelligence, with the aim of identifying key challenges in the transmission, collection, and processing of PNR data. Based on the outcomes of the survey, the present report also provides a list of recommendations that could pave the future of the processing of PNR data by enhancing the capabilities of the PIUs. As such, the document seeks to contribute to the existing literature concerning travel intelligence within the European Union, which currently remains somewhat limited in scope and depth.

## Methods

In the context of the TENACITy project, it was considered necessary to deliver an in-depth analysis of the current state of play of the processing of PNR data in European countries. To do so, the Centre for Security Studies (KEMEA) provided an analysis - as presented in this article - based on a survey that took the form of a questionnaire and was circulated, from early December 2023 to early February 2024, through the EU Survey platform, to all PIUs participating in the TENACITy consortium and the EU PIUs. Fourteen PIUs replied to the survey ^5^. The participation to the survey was anonymous in order to protect the privacy and personal data of the respondent, whose role within their PIU varied from analysts, shift leaders, carrier connectivity experts, operators to officers, heads of the PIUs, and directors. Unclear or missing information were not taken into consideration. Due to the 'anonymous' nature of the survey, the researchers were not able to contact the respondents for any clarification needed. The limited access to participants is a common hurdle when the research targets security actors ( Glouftsios & Leese, 2023). While the findings presented herein reflect the opinions of the surveyed PIUs, it is noteworthy to stress that, yet upon identifying challenges, the participating PIUs within the TENACITy project affirmed the presence of similar issues among their counterparts in other nations. Furthermore, the survey results broadly align with the report on the review of PNR Directive ( European Commission, 2020b), which takes into consideration all EU PIUs.

Inclusion criteria for the review encompass research studies, reports, and evaluations specifically focused on the implementation of the PNR Directive and Passenger Information Units (PIUs), incorporating both qualitative and quantitative methodologies such as surveys, interviews, case studies, and observational studies. Systematic reviews and meta-analyses that provide data on PIUs are also included. Studies must be published in English, or in other languages if translation resources are available. Exclusion criteria eliminate editorials, opinion pieces, and narrative reviews lacking empirical data on PIUs, as well as case reports and case series unrelated to the functioning or impact of PIUs. Additionally, studies with significant methodological flaws or a high risk of bias, those not involving users or stakeholders of PIUs, and studies published in languages without available translation resources are excluded.

More specifically, the survey aimed at the identification of i) the level of implementation of the technical solutions to process PNR and API data, ii) the technical solution developed, and iii) the user and system requirements and challenges faced by PIUs. Questionnaires have been widely used to collect data from stakeholders and thus they constitute the backbone of any survey ( Roopa & Rani, 2012). Although questionnaires are usually used to collect primary quantitative data ( Reja *et al.*, 2003), the present survey targeted the extraction of both quantitative and qualitative data. The data collected from the survey were analysed to provide a thorough picture of the state-of-play of the PIUs. The data collected related to the challenges faced by the PIUs were analysed and resulted into the identification of six areas of challenges: i) data transmission, ii) collection of data, iii) data quality, iv) data analysis, v) staff matters, and vi) legal matters. Each category of challenges integrates various gaps that are presented in the following section.

## Limitations

One significant limitation of this study is the absence of a formal risk of bias assessment. No standardised tools were used, and no reviewers were assigned to independently evaluate bias. Readers should interpret the results with caution, acknowledging that unidentified biases in the included studies could influence the overall conclusions. Future research should incorporate comprehensive bias assessment methods to enhance the robustness of findings.

## Results

The data collected from the survey were analysed to identify key areas that PIUs find challenging or problematic. Most of the challenges were identified throughout the process of the data collected and handled by the PIUs, that is from the data transfer phase to the data collection, the data analysis, and the data quality. Organisational and internal problems as well as aspects of a legal nature were also identified as challenging by the PIUs.

### Data transfer

One of the most important challenges the PIUs are facing is related to the field of data transmission. Under the Directive, the custodians of the PNR data are the PIUs to which the air carriers shall transfer PNR data. Following their processing, the PIUs may transfer PNR data or the results of their analysis to the national competent authorities, to the PIUs of other Member States, and to Europol ( The European Parliament & the Council of the European Union, 2016a). On a case-by- case basis Member States may also exchange PNR data or the processing results with third countries.

From the early 1990’s inception of collecting passengers’ data to use them in the fight against terrorism, the need of following a standardised approach became evident ( World Customs Organisation, 2015). Understanding the gravity of the risks, the WCO, IATA, and afterwards ICAO developed a series of standards to support advance submission of passenger data. Amongst others, the WCO/IATA/ICAO ^6^ standards offer guidelines for the transmission of PNR data from air carriers to the PIUs. Similarly, the implementation of the EU PNR Directive and the establishment of common protocols and formats in order for air carries to transfer PNR data to PIUs ( European Commission, 2017) follows the same approach. Indeed, the implementation of a standardised procedure for data transmission has multiple benefits for both the air carriers and the PIUs. These benefits include faster data analysis and checking across the various databases, improved compliance by air carriers, lower costs for both airline companies and governments, and system harmonisation ( World Customs Organisation (2015).

However, the survey showed that, even with the existing standards, the PIUs are facing data standardisation issues. First of all, it should not be taken for granted that all the actors involved in the PNR/API data transfer implement existing standards. According to the survey, some air carriers may not support automated transmissions of PNR data, making it challenging for PIUs to collect comprehensive datasets. This lack of data from air carriers undermines the effectiveness of security measures and compromises the ability to identify unknown threats. Secondly, the EDIFACT PNRGOV ( International Air Transport Association, 2016), which is the standard for PNR data transmission, is a standard message and not a standard content, which implies difficulties in managing them due to possible variations in the format or duplications ( World Customs Organisation, 2015). As such, these related to data transmission challenges have implications on both the collection and quality of the data.

As above mentioned, according to the Directive, the PIUs shall be responsible for i) the PNR data collection from air carriers, their process and transferring of those data or processing results to the competent authorities, and ii) the exchange of those data and processing results with other EU PIUs and with Europol. Yet, challenges arise in both processes. Regarding the transmission of PNR data to NCAs, several respondents mentioned that problems arise as some Member States do not obtain secure channels to transmit them.

Regarding the exchange of data from PIU to PIU, the Directive identifies two cases: i) a PIU may transfer data on its own initiative to another PIU, or ii) after receiving a request from another PIU. In the formed case, in which a PIU spontaneously transferred PNR data to another PIU, were limited, with a significant number of Member States to have never spontaneously transferred data to another EU PIU ( European Commission, 2020b). As for the latter case, the analysis of the survey results pointed out the challenge of the increasing number of requests, and thus, the increasing workload of the PIUs. Specifically, the practice of sending broad and unspecified requests to more than one PIU was highlighted together with the need of the PIUs to check the data and information collected against databases ahead of sending a request to another PIU. In addition to that, the respondents to the survey indicated that not all PIUs are using the agreed PIU-PIU template to request data, which has been used by the majority of Member States in an effort to facilitate the exchange of PNR data (ibid.).

### Collection of data

The PIUs are becoming more and more operational and some, being in a more advanced position than others, mentioned that they often lack the necessary technological infrastructure to efficiently handle the vast amount of PNR data generated by air travel. The volume of data generated can overwhelm outdated systems, leading to delays and inefficiencies in processing critical information. PIUs may lack access to advanced technologies that facilitate large-scale data analysis. Without access to tools such as machine learning algorithms and big data analytics platforms, PIUs struggle to effectively process and analyse the vast amount of PNR data at their disposal.

Moreover, the acquisition of PNR data from travel agencies presents significant challenges attributable to a multitude of logistical and legal impediments. Non-carrier economic entities, notably travel agencies and tour operators, may frequently deviate from the standardised data formats typically adhered to by air carriers. Consequently, this variance often culminates in incomplete datasets, thereby impeding the thoroughness of threat assessments.

According to Article 6 par. 2 of the Directive during the passengers’ assessment before their arrival or departure (a), The PIUs have the mandate to compare PNR data against databases on the grounds of terrorism and serious crime prevention, detection, investigation, and prosecution, always in line with existing EU, national, and international rules ( The European Parliament & the Council of the European Union, 2016a). However, from the replies received, the PIUs mentioned that they often face difficulties in establishing efficient connectivity with other databases, limiting their ability to cross-reference PNR data with other relevant information sources. This lack of integration hampers the effectiveness of security measures and compromises the ability to detect potential threats.

Lastly, the establishment of a reliable link between new and historical flights of the same individual poses a significant challenge for PIUs. Without efficient mechanisms in place to track individuals across different flights, identifying patterns of suspicious behaviour becomes increasingly difficult.

### Data quality

Another major challenge the PIUs are facing is that of the poor data quality. The quality of the data collected by the PIUs refers to representativeness, completeness, accuracy, and consistency of the data. The PIUs have to deal with the poor quality of data for various reasons. Usually, the passengers book their airline tickets on their own or through a travel agency. The respondents to the survey indicated regular ‘misspellings’ as an important issue which affects the data accuracy and completeness. In most cases misspellings are not done intentionally by the passengers or the travel agents, in order to hide a criminal action ( Glouftsios & Leese (2023)).

Regarding the completeness of data, the optional nature of certain passenger data also impacts data quality. It should be clarified that airline companies are not legally bοund to validate the data they are collecting. ΑΝΝΕΧ I of the Directive lists the data that, if collected by an air carrier in the normal course of their business, they should be transmitted to the PIUs, data like the dates of travel and travel itinerary, ticket information, contact details, travel agent, payment information, seat number, and baggage information are collected ( European Commission, n.d.). Based on the findings of the survey and review report on the implementation of the Directive ( European Commission, 2020a), it has been underscored that certain data categories must be mandated for collection by air carriers, with date of birth being deemed the most important. Furthermore, in addition to non-mandatory data collected, the absence of validation procedures prior to their acquisition from PIUs introduces inaccuracies. For instance, it is prevalent for travel agents to provide their contact details instead of those of the passengers during ticket booking. Yet, based on the answers received, even the data quality check conducted by the PIUs is challenging for them, as it has to be performed manually. The absence of automated technological solutions to monitor the validity and quality of the data received causes a huge workload for the PIU personnel.

To face the challenge of poor data quality, several PIUs cross-validate PNR data with data and information from other sources, most notable the API data, when applicable ( Glouftsios & Leese, 2023). Even though, the API data are considered more accurate, their collection is not mandatory for intra-Schengen flights, and the fact that ID’s checks are not always conducted at the airports, diminish the protentional of leveraging from the cross match between API and PNR data. Against this background, the possible adoption of the EU Regulation ( European Commission, 2022) 2019/818, is anticipated to address several data quality issues encountered by the PIUs and thus minimise the security gaps that they are currently facing with.

### Data analysis

Another factor that impacts both the quality and the subsequent analysis of the data collected is that of different PNRs for the same trip. Even though the Directive includes the ‘split/divided PNR information’ in the PNR data, it does not include the ‘linked PNR information’. The ‘split/divided PNR information’ refers to the process of dividing a PNR record locator into two or more, so as to handle the reservation separately, while the linked PNR is the process of connecting a PNR locator with another PNR locator. The latter is usually conducted upon request from the passenger or the travel agent in order for the passengers to sit together in the plane. Apart from that, it is possible for travel agents and passengers that are flying together to book separate tickets and not link them at all. While seemingly innocuous, this practice may obscure vital information from PIUs.

The capabilities of the PIUs are also affected by the practice of ‘broken travels’. Broken travels include multi-stage routes that combine many different modes of transport and is a technique highly preferred by terrorists for entering EU ( Hornak, 2015). By collecting and handling passengers’ data only from air carriers, the PIUs can have only a limited intelligence picture of the cross-border movements, which constitutes an important security gap.

### Staff matters

Since the adoption of the Directive ( The European Parliament and the Council of the European Union, 2016a), there has been a notable increase in the operational functionality of EU PIUs leading to one significant challenge, which is the efficient management of the vast amount of PNR data generated by air travel. The operational efficiency of PIUs is influenced by various factors, such as technological advancements and staff training. While some units have made significant strides in these areas, others lag behind due to resource constraints or organisational limitations. At the same time, several PIUs highlighted the need for trainings in data analysis, which is crucial for extracting insights from PNR data in order to also develop robust targeting rules. Without proper training, staff may struggle to effectively utilise available resources, leading to gaps in security.

### Legal matters

Lack of reciprocity with third countries further complicates efforts to effectively process PNR data. As also mentioned in the Staff Working Document on Review of the Directive ( European Commission, 2020b), on the one hand, the intensification of requests for PNR data from third countries, not having a respective PNR Agreement with the EU in force, has led in some cases to the appliance of retaliatory measures from certain non-EU countries. For instance, reports indicate that certain third countries have directed their national airlines to cease PNR data transfers to the EU Member States, thereby affecting the implementation of the Directive and creating security risks. On the other hand, air carriers may face conflicts of laws between EU data protection regulations and requirements from destination countries, potentially resulting in sanctions. It was thus considered imperative, from several PIUs, to introduce a comprehensive international cooperation strategy to facilitate the transfer of PNR data to and from EU countries, fully respecting fundamental rights. Rulings of the Court of Justice of the European Union (CJEU) may introduce additional complexities and constraints for PIUs. Under the Directive, PNR data can be retained for a period of five years and must be depersonalised after a period of six months. However, the CJEU in its Judgment in Case C-817/19 ruled that beyond the initial six-month e retention period, of PNR data is not strictly necessary unless there is objective evidence – such as a verified positive match during the advance assessment – that indicates a risk to terrorist offences or serious crime linked to the passengers’ air travel. Conversely, the Court deemed that the PNR data of all air passengers within the initial six-month period generally does not exceed what is strictly necessary’ ( Court of Justice of the European Union, 2022). Adapting to new legal requirements while ensuring compliance with existing regulations can pose significant challenges for PIUs, impacting their ability to effectively process PNR data and ensure passenger safety.

## Recommendations

Although it is the PIUs that have mainly to deal with the above-mentioned challenges, their resolution requires a collaborative effort amongst policymakers, security practitioners, and industry to develop and promote innovation. Notably, technological advancements can enhance the operational capabilities of the PIUs, but their deployment is not sufficient to solve core problems. Based on the results of the survey, the present section presents a series of recommendations to fill the capability gaps of the PIUs that pertain to the technological, political, legal sphere.

### Validation of the data quality prior to their transmission to the PIUs

As previously described, the air carriers are not responsible for the validation of the data provided by the passengers. This implies that the passengers may provide information that is not verified until their collection from the PIUs, causing informational gaps and operational obstacles. An effective validation mechanism could enhance the data quality without causing additional cost to the air carriers. This mechanism shall have at its core tools that automatically validate the quality of the data and in case of mistakes, misspellings, or missing data do not allow the passenger to move to the booking of an airline ticket. In combination with the automatic verification of the contact details provided in the ticket reservation, this mechanism could significantly enhance the PIUs capabilities. The new provisional agreement on air passenger data moves towards this direction as establishes the automated data-collection and verification mechanisms to guarantee the accuracy of the data ( The Council of the European Union, 2024).

### Automation of data quality controls

Upon the data collection by the PIUs, the validation of their quality is of paramount importance. Some survey participants mentioned some tools that conduct data quality controls, but they are insufficient. This lack of automated data quality controls to check for possible errors, misspellings, invalid data poses a huge workload to the PIUs which they have to check manually the data transmitted by the air carriers. The development and deployment of tools that can automatically validate the data quality and detect mistakes or fields that have not been filled correctly could minimise the heavy workload of the under-staffed PIUs. In a subsequent step, the detected errors could be used to generate error reports per air carrier so as to inform the latter respectively.

### The development of automated tools that will generate rule suggestions based on previous hits

A recommendation stemming from the development of automated tools generating rule suggestions based on previous hits is to prioritise the refinement and enhancement of these automated systems. By leveraging advanced machine learning algorithms and data analytics, these tools can analyse historical hits or encounters, as well as feedback and validation from the PIU’s operators, thereby generating more accurate and effective targeting rule suggestions over time. The establishment of mechanisms for real-time feedback and validation from the operators will be essential to ensure the relevance and reliability of the generated rule suggestions. Those suggestions, that will include detailed explanations on the utilized criteria and relevant weights, could then be manually reviewed and potentially fine-tuned by the PIU experts and analysts and transformed into actual rules, that could be activated upon successful evaluation for a given time period. This process of refinement will not only enhance the efficiency and effectiveness of targeting efforts but also enable the adaptation to evolving threats and changing operational environments.

### Standardisation of data transfers to third countries and to Competent Authorities (CA)

In order to enhance data transfers between PIUs and third countries, several steps can be taken. Firstly, leveraging EU Security Research initiatives is suggested as effective starting points for involving PIUs and standardizing data sharing practices. However, it is emphasized that additional funding is imperative to sustain and expand such endeavours. Secondly, there is a consensus among multiple PIUs on the necessity to establish uniform and secure channels for data exchange, particularly with third countries and NCAs. While some PIUs currently rely on email for data transfer, many advocate for the implementation of automated solutions to streamline this process. Thirdly, it has been proposed to revise existing data exchange methods, such as the excel template utilised for sharing passengers' data via Europol’s SIENA message system ( Europol, 2022). Suggestions include incorporating drop-down menus to minimize errors and enhance efficiency in data transmission. These recommendations aim to facilitate smoother and more secure data transfers, thereby improving collaboration and information exchange among relevant stakeholders.

### Standardisation of transfers of data from non-carrier economic operators

The operational value of the collection of the information from non-carrier economic operators, like tour operators and travel agencies was highlighted in the review of the implementation of the Directive ( European Commission, 2020a). A recommendation for the standardisation of transfers of data from non-carrier economic operators is to establish clear and uniform protocols and guidelines for data transfer processes. This entails developing standardised formats, protocols, and encryption methods to ensure secure and efficient transmission of data between non-carrier economic operators and PIUs. Additionally, providing comprehensive training and support to non-carrier economic operators on data protection regulations, privacy safeguards, and compliance requirements will be crucial to ensure adherence to established standards, EU, and national legislation. By promoting consistency and coherence in data transfer procedures, standardization efforts can enhance interoperability, streamline operations, and mitigate potential risks associated with data breaches or inconsistencies, ultimately contributing to more effective and harmonized security measures across borders.

### The collection of specific passenger’s data elements shall become mandatory

In light of the current voluntary nature of the collection of PNR data as outlined in Annex I of the Directive and considering that air carriers are expected to transfer data collected in the normal course of their business, exists a potential challenge regarding data availability. To address this, it is recommended that certain key data elements are made mandatory for collection by air carriers. These mandatory data fields should include but not be limited to: date of birth, contact details, API, payment method, date of booking, final confirmation on the boarding status of passengers indicating whether they have boarded or not, itinerary details, information regarding travel companions when applicable, and document information, including number, issuance country, expiration date, and citizenship. Ensuring the mandatory collection of these data elements would enable analysts to have a comprehensive dataset necessary for conducting thorough assessments. This recommendation is crucial for enhancing the overall quality and completeness of the data available for analysis and decision-making within the aviation security domain.

### Expansion of the PIUs’ mandate to other modes of transport

It is recommended that the European Union and its Member States adopt a unified and coordinated approach towards the expansion of travel data collection beyond air carriers to include other modes of transport such as maritime, trains and buses, in full compliance with the principle of proportionality and necessity. This holistic approach is essential for enhancing cross-border cooperation in combating terrorism and serious crime, as it ensures security measures cover multiple transportation modes, reducing the potential for individuals to exploit gaps in information sharing. By extending collaboration among PIUs to encompass various modes of transport, security analysts can obtain a more comprehensive view of a traveller's journey, thereby minimising operational gaps posed by "broken travels" and indirect routes. Additionally, this unified approach streamlines efforts, reduces duplication of resources, and fosters public confidence in passenger safety and security. Towards this direction, ( The Council of the European Union, 2019) to explore the necessity and feasibility of collecting, storing, and processing of PNR data from cross-border forms of transport other than air traffic the Council of the European Union, is moving towards the right direction has already made recommendations, indicating progress in the right direction.

## Conclusions

The survey conducted in the context of the TENACITy project showed the diverse nature of the challenges that accompany all the phases of the PNR data processing, from the transfer phase to the collection, and process phase. The lack of commonly used standards by all the involved stakeholders and the poor data quality can be placed at the base of the challenges faced by the PIUs that cause a ‘domino effect’ to their operational capabilities. Consequently, there is a collective desire for certain data elements to be mandated for collection by air carriers, such as passengers' dates of birth. It is noteworthy though that the nature of the challenge may differ from the nature of a possible solution. For example, most of the PIUs participated to the survey stated that the existing technological tools cover a big part of their operational needs, yet a possible solution to the lack of personnel to deal with the heavy workload, could be the development of automated tools to perform quality controls to the PNR data received. Moreover, while some PIUs are currently processing PNR data from carriers lacking automated transmission capabilities, the availability of datasets for comparison remains limited. This challenge extends to PIUs collecting PNR data from other modes of transport.

The Directive has marked air travel intelligence in the EU and put the PIUs in the centre of it. Its review showed the significant added value of their creation and operation in the field of travel intelligence, and crime and terrorist prevention. Therefore, it is important to explore the ways on how they can be evolved in the future and by extension to further support travel intelligence strategies. The identification of key challenges confronting PIUs serves as an initial step in this trajectory, paving the way for informed enhancements and ensuring the continued efficacy of travel intelligence initiatives in full respect of fundamental rights.

## Consent to participate

All participants provided written informed consent by signing an information sheet and consent form for participation in the survey conducted as part of the TENACITy project.

## Data Availability

The sensitive nature of the TENACITy project necessitates strict restrictions on the disclosure of survey data. Making the survey data openly available poses significant security and privacy risks, as it could potentially compromise the confidentiality of respondents and reveal sensitive operational details of Passenger Information Units. Access to anonymised survey data will be granted on a case-by-case basis, subject to the approval of the Security Advisory Board of the TENACITy project. Interested readers and reviewers may request access by contacting the authors via email. Zenodo: TENACITy, Navigating the challenges of passenger name record data and the way forward DOI: https://doi.org/10.5281/zenodo.12743231 Aposkiti, C., & Makri, F. (2024). This project contains the following underlying data: Questionnaire Creative Commons Attribution 4.0 International: https://creativecommons.org/licenses/by/4.0/

## References

[ref-1] Aposkiti C Makri F : Navigating the challenges of passenger name record data and the way forward. *Zenodo.* 2024. 10.5281/zenodo.12743231 PMC12395126 40894387

[ref-2] Court of Justice of the European Union: CJEU Press Release No 105/22: (21 June 2022). The Court considers that respect for fundamental rights requires that the powers provided for by the PNR Directive be limited to what is strictly necessary. Judgment of the Court in Case C-817/19 | Ligue des droits humains. Court of Justice of the European Union, Luxemburg,2022. Reference Source

[ref-3] De Hert P Papakonstantinou V : The EU PNR framework decision proposal: towards completion of the PNR processing scene in Europe. *Comput Law Secur Rev.* 2010;26(4):368–376. 10.1016/j.clsr.2010.05.008

[ref-4] European Commission: Commission Implementing Decision (EU) 2017/759 of 28 April 2017 on the common protocols and data formats to be used by air carriers when transferring PNR data to Passenger Information Units C/2017/2743 OJ L 113, 29.4.2017. Official Journal of the European Union,2017;48–51. Reference Source

[ref-5] European Commission: 128 final Report from the Commission to the European Parliament and the Council on the review of Directive 2016/681 on the use of passenger name record (PNR) data for the prevention, detection, investigation and prosecution of terrorist offences and serious crime {SWD (2020) 128 final}. Brussels,2020a. Reference Source

[ref-6] European Commission: Passenger data. Migration and home affairs. (n.d.). Reference Source

[ref-7] European Commission: 128 final commission staff working document accompanying the document report from the commission to the european parliament and the council on the review of Directive 2016/681 on the use of Passenger Name Record (PNR) data for the prevention, detection, investigation and prosecution of terrorist offences and serious crime {COM (2020) 305 final}. Brussels,2020b. Reference Source

[ref-8] European Commission: Proposal for a regulation of the european parliament and of the council on the collection and transfer of advance passenger information for the prevention, detection, investigation and prosecution of terrorist offences and serious crime, and amending Regulation (EU) 2019/818 COM(2022)731 final. Strasburg,2022. Reference Source

[ref-9] Europol: Secure Information Exchange Network Application (SIENA): ensuring the secure exchange of sensitive and restricted information.2022. Reference Source

[ref-10] Glouftsios G Leese M : Epistemic fusion: passenger information units in the making of international security. *Rev Int Stud.* 2023;49(1):125–142. 10.1017/S0260210522000365

[ref-11] Hornak L : ‘Broken travel’ keep ISIS’ western recruits off the radar of intelligence agencies. *The World.* 2015. Reference Source

[ref-12] International Air Transport Association: Passenger and airport data interchange standards xml implementation guide - PNRGOV (version 16.1).2016. Reference Source

[ref-13] Kanellopoulos AN : Travel intelligence as a tool for counterintelligence and border security. *Security and Defence Quarterly.* 2024;45(1):55–67. 10.35467/sdq/174523

[ref-14] Reja U Manfreda KL Hlebec V : Open-ended vs. close-ended questions in web questionnaires. *Developments in Applied Statistics.* 2003;159–177. Reference Source

[ref-15] Roopa S Rani M : Questionnaire design for a survey. *J Indian Orthod Soc.* 2012;46(4):273–277. 10.5005/jp-journals-10021-1104

[ref-16] The Council of the European Union: Council Directive 2004/82/EC of 29 April 2004 on the obligation of carriers to communicate passenger data OJ L 261, 6.8.2004. Official Journal of the European Union,2004;24–27. Reference Source

[ref-17] The Council of the European Union: Council conclusions on widening the scope of the use of Passenger Name Record (PNR) data to forms of transport other than air traffic. Council conclusions (2 December 2019), Brussels. 14746/19.2019. Reference Source

[ref-18] The Council of the European Union: Air passenger data: council and the european parliament reach provisional agreement to increase security and enhance border management [Press release].2024. Reference Source

[ref-19] The European Parliament and the Council of the European Union: Directive (EU) 2016/681 of the European Parliament and of the Council of 27 April 2016 on the use of passenger name record (PNR) data for the prevention, detection, investigation and prosecution of terrorist offences and serious crime. Official Journal of the European Union. OJ L 119, 4.5.2016.2016a;132–149. Reference Source

[ref-20] The European Parliament and the Council of the European Union: Regulation (EU) 2016/399 of the European Parliament and of the Council of 9 March 2016 on a Union Code on the rules governing the movement of persons across borders (Schengen Borders Code) (codification). Official Journal of the European Union,2016b. Reference Source

[ref-21] US Government Publishing Office: Aviation and transportation security act: to improve aviation security and for other purposes. Washington, D.C. S.1447 - 107th US Congress.2001. Reference Source

[ref-22] World Customs Organisation: Challenges and opportunities of passenger data systems.2015. Reference Source

