# Navigating the challenges of passenger name record data and the way forward

**DOI:** 10.12688/openreseurope.18037.2

**Published:** 2025-05-19

**Authors:** Christiana Aposkiti, Freideriki Makri

**Affiliations:** 1 P.Kanellopoulou Str.4, KEMEA, Center for Security Studies, Athens, 10177, Greece; 2 P.Kanellopoulou Str.4, Hellenic Police, Athens, 10177, Greece

**Keywords:** PIUS Challenges, Travel Intelligence Recommendations, Passenger Name Record (PNR), Passenger Information Units (PIUs), counterterrorism, crime prevention, EU PNR Directive, Advance Passenger Information (API)

## Abstract

**Background:**

The TENACITy EU-funded project investigates the multifaceted challenges surrounding the utilisation of Passenger Name Record (PNR) data following the implementation of the PNR Directive (EU) 2016/681, which marks a significant shift in EU air travel intelligence.

**Methods:**

This study employed a combination of research and survey methodologies to gather data from various stakeholders involved in the implementation of the PNR Directive. The survey focused on identifying the key obstacles faced by Passenger Information Units (PIUs), including the absence of standardised practices and issues of data quality.

**Results:**

The findings highlight two primary obstacles confronting PIUs: the lack of standardised practices among stakeholders and the poor quality of PNR data. Additionally, fragmented implementation and certain regulatory barriers were identified as factors that present challenges to the optimal utilisation of PNR data for counterterrorism and crime prevention efforts.

**Conclusions:**

Addressing these challenges requires nuanced solutions, with technological tools presenting potential remedies to operational constraints. There is a collective call for mandating specific data elements to enhance the effectiveness of PNR data utilisation. This paper provides insights and recommendations to enhance PIUs' capabilities, contributing to the ongoing discourse on EU travel intelligence.

## Introduction

The 9/11 attack signified a new era on the field of travel intelligence which refers to ‘the systematic collection, analysis, and utilisation of travel-related information and intelligence to enhance security measures and law enforcement efforts’ ( Kanellopoulos, 2024) since it highlighted the importance of the processing of Passenger Name Record (PNR) data, which prior to the attack were mainly collected by the air carriers in a non-systematic way to book air travel reservations, to fight and even prevent terrorism and organised crime ( De Hert & Papakonstantinou, 2010). The attack led to the implementation of the Aviation and Transportation Security Act in 2001 by the United States ( US Government Publishing Office, 2001), which consequently opened the path of the air travel intelligence landscape as it is known today.

A few years later, the terrorist attack in Madrid in 2004 initiated the European Union’s (EU) more active role in the fight against terrorism, as it adopted a stricter policy for the protection of its external borders. The EU’s policy towards the prevention of terrorist attacks is underpinned by the implementation of the Advance Passenger Information (API) Directive ( The Council of the European Union, 2004), and the Schengen Borders Code ( The European Parliament & the Council of the European Union, 2016b), that foster the information and intelligence exchange with third countries in relation to the cross-border movement of people. The EU has undertaken additional efforts in border management embodying a multifaceted approach to safeguarding its borders and combating terrorism and serious cross border crime that complement the API Directive. More recently, these efforts include the Entry Exit System (EES) ^1^ and the European Travel Information and Authorisation System (ETIAS) which are not still in operation ^2^.

In this context, the use of PNR data has been integral to the international cooperation efforts of the EU against terrorism and serious crime for nearly two decades with the implementation of the Passenger Name Records (PNR) Directive (EU) 2016/681 (henceforth “the Directive”) being the cornerstone of them. The Directive regulates the use of PNR data in the European Union for the prevention, detention, investigation and prosecution of terrorist offences and serious crime and calls for each Member State to establish a Passenger Information Unit (PIU) ( The European Parliament & the Council of the EU, 2016a). The PIU serves as the sole authority for collecting, storing, processing, and transferring under specific conditions PNR and API data ^3^ to other PIUs, National Competent Authorities (NCAs), Europol, and third countries. This process must fully respect fundamental rights, including privacy and personal data protection.

The European Commission's Review of the Directive ( European Commission, 2020b) highlights the increasing recognition globally, not limited to EU Member States, of the utility of PNR data as a tool for law enforcement in the fight against terrorism and serious crime. Yet, the findings also showed that the implementation of the Directive faces delays and fragmentation. Data quality and accuracy were considered paramount for improving the collection of relevant data used to combat terrorism and serious crime. Despite EU legislation, variations in perception and methodologies in implementing the Directive were reported. PIUs officers outlined incomplete, inaccurate, or untimely data entry, while the ‘broad’ and ‘relatively unclear formulation’ of the Directive was considered the main reason why PIUs did not move to spontaneous transfers ( European Commission, 2020a).

In this complex travel intelligence landscape, the Travel Intelligence Against Crime and Terrorism - TENACITy project ^4^ envisions to address key challenges by developing innovative tools, offering trainings, and establishing a holistic approach to prevent and fight terrorism and crime through travel intelligence. The present document reports on the findings of a survey conducted in the context of the TENACITy project, that was addressed to the PIUs, one of the main actors of today’s travel intelligence, with the aim of identifying key challenges in the transmission, collection, and processing of PNR data. Based on the outcomes of the survey, the present report also provides a list of recommendations that could pave the future of the processing of PNR data by enhancing the capabilities of the PIUs. As such, the document seeks to contribute to the existing literature concerning travel intelligence within the European Union, which currently remains somewhat limited in scope and depth. While this article aspires to provide insights on the operational effectiveness of the PNR directive, it does not move to a comprehensive analysis of the various historical and political aspects that are related to the adoption and implementation of the directive.

## Methods

In the context of the TENACITy project, it was considered necessary to deliver an in-depth analysis of the current state of play of the processing of PNR data in European countries. To do so, the Centre for Security Studies (KEMEA) provided an analysis - as presented in this article - based on a survey that took the form of a questionnaire and was circulated, from early December 2023 to early February 2024, through the EU Survey platform, to all PIUs participating in the TENACITy consortium and the EU PIUs. Fourteen PIUs replied to the survey ^5^. The participation to the survey was anonymous in order to protect the privacy and personal data of the respondent, whose role within their PIU varied from analysts, shift leaders, carrier connectivity experts, operators to officers, heads of the PIUs, and directors. Unclear or missing information were not taken into consideration. Due to the 'anonymous' nature of the survey, the researchers were not able to contact the respondents for any clarification needed. The limited access to participants is a common hurdle when the research targets security actors ( Glouftsios & Leese, 2023). While the findings presented herein reflect the opinions of the surveyed PIUs, it is noteworthy to stress that, yet upon identifying challenges, the participating PIUs within the TENACITy project affirmed the presence of similar issues among their counterparts in other nations. Furthermore, the survey results broadly align with the report on the review of PNR Directive ( European Commission, 2020b), which takes into consideration all EU PIUs.

Inclusion criteria for the review encompass research studies, reports, and evaluations specifically focused on the implementation of the PNR Directive and Passenger Information Units (PIUs), incorporating both qualitative and quantitative methodologies such as surveys, interviews, case studies, and observational studies. Systematic reviews and meta-analyses that provide data on PIUs are also included. Studies must be published in English, or in other languages if translation resources are available. The article’s primary scope is to present the results of a survey with EU PIUs in the context of the TENACITy project, along with some findings occurred from their analysis. Although existing literature has been used, and cited accordingly, to support the analysis of the results, the article does not include an exhaustive review of the relevant literature.

More specifically, the survey aimed at the identification of i) the level of implementation of the technical solutions to process PNR and API data, ii) the technical solution developed, and iii) the user and system requirements and challenges faced by PIUs. Questionnaires have been widely used to collect data from stakeholders and thus they constitute the backbone of any survey ( Roopa & Rani, 2012). Although questionnaires are usually used to collect primary quantitative data ( Reja *et al.*, 2003), the present survey targeted the extraction of both quantitative and qualitative data. In this respect, the survey consisted of sixteen (16) open and closed questions. The quantitative data were analysed by using graphs, that can be found in the ‘Extended data’ section.

The data collected from the survey were analysed to provide a thorough picture of the state-of-play of the PIUs.The data collected related to the challenges faced by the PIUs were analysed and resulted into the identification of six areas of challenges: i) data transmission, ii) collection of data, iii) data quality, iv) data analysis, v) staff matters, and vi) legal matters. Each category of challenges integrates various gaps that are presented in the following section.

### Limitations

Several limitations must be acknowledged. To start with, despite the sample size offering a diverse mix of roles and functions, it can be considered as relatively small (14 PIUs). In addition, the anonymous nature of the survey, restricted the authors’ ability to seek any clarifications and follow up questions. Still, receiving replies from a broad range of positions, from analysts to directors, gave a broad view of the operational and technical issues the PIUs are facing. Moreover, confidentiality prevents the authors from mentioning explicitly which responses came from EU or third-country PIUs, yet it must be noted that the challenges that are referred to in this article appear to be widely shared across various PIUs.

Second, one significant limitation of this study is the absence of a formal risk of bias assessment. No standardised tools were used, and no reviewers were assigned to independently evaluate bias. Readers should approach the results with caution, acknowledging that unidentified biases in the included studies could influence the overall conclusions. To strengthen future research, it would be helpful to use more thorough methods for assessing bias, to enhance the robustness of findings. Future research would also benefit from including interviews or workshops with PIU representatives to explore these topics in more depth.

## Results

The current section concentrates on the challenges and issues PIUs are tackling while implementing the PNR directive, without providing a comprehensive analysis of the relevant regulations that frame their function. Most of the challenges were identified throughout the process of the data collected and handled by the PIUs, that is from the data transfer phase to the data collection, the data analysis, and the data quality. Apart from operational issues, the participants to the survey identified also technological, procedural, organisational and internal problems as well as several aspects of a legal nature. All of the challenges listed below are based on the survey results and occasionally are complemented with existing literature findings.

### Data transfer

One of the most important challenges the PIUs are facing is related to the field of data transmission. Under the Directive, the custodians of the PNR data are the PIUs to which the air carriers shall transfer PNR data. Following their processing, the PIUs may transfer PNR data or the results of their analysis to the national competent authorities, to the PIUs of other Member States, and to Europol ( The European Parliament & the Council of the European Union, 2016a). On a case-by- case basis Member States may also exchange PNR data or the processing results with third countries.

From the early 1990’s inception of collecting passengers’ data to use them in the fight against terrorism, the need of following a standardised approach became evident ( World Customs Organisation, 2015). Understanding the gravity of the risks, the World Customs Organisation (WCO), the International Air Transport Association (IATA), and afterwards the International Civil Aviation Organisation (ICAO) developed a series of standards to support advance submission of passenger data. Amongst others, the WCO/IATA/ICAO ^6^ standards offer guidelines for the transmission of PNR data from air carriers to the PIUs. Similarly, the implementation of the EU PNR Directive and the establishment of common protocols and formats in order for air carries to transfer PNR data to PIUs ( European Commission, 2017) follows the same approach. Indeed, the implementation of a standardised procedure for data transmission has multiple benefits for both the air carriers and the PIUs. These benefits include faster data analysis and checking across the various databases, improved compliance by air carriers, lower costs for both airline companies and governments, and system harmonisation ( World Customs Organisation (2015).

However, the survey showed that, even with the existing standards, the PIUs are facing data standardisation issues. First of all, it should not be taken for granted that all the actors involved in the PNR/API data transfer implement existing standards. According to the survey, some air carriers may not support automated transmissions of PNR data, which makes the data collection and data management process more complicated. The lack of automated means does not necessarily lead to a complete lack of data, but it may result in delays, errors, or data of low quality, which consequently affect the PIUs’ effectiveness and operational capabilities to identify threats. Secondly, the EDIFACT PNRGOV ( International Air Transport Association, 2016), which is the standard for PNR data transmission, is a standard message and not a standard content, which implies difficulties in managing them due to possible variations in the format or duplications ( World Customs Organisation, 2015). As such, these related to data transmission challenges have implications on both the collection and quality of the data. While these issues do not necessarily amount to non-compliance with the EU PNR Directive, they highlight operational gaps and the need for rigorous mechanisms that will ensure the common application of standards across all stakeholders.

As above mentioned, according to the Directive, the PIUs shall be responsible for i) the PNR data collection from air carriers, their process and transferring of those data or processing results to the competent authorities, and ii) the exchange of those data and processing results with other EU PIUs and with Europol. Yet, challenges arise in both processes. Regarding the transmission of PNR data to NCAs, several respondents mentioned that problems arise as some Member States do not obtain secure channels to transmit them.

Regarding the exchange of data from PIU to PIU, the Directive identifies two cases: i) a PIU may transfer data on its own initiative to another PIU, or ii) after receiving a request from another PIU. In the formed case, in which a PIU spontaneously transferred PNR data to another PIU, were limited, with a significant number of Member States to have never spontaneously transferred data to another EU PIU ( European Commission, 2020b). As for the latter case, the analysis of the survey results pointed out the challenge of the increasing number of requests, and thus, the increasing workload of the PIUs. Specifically, the practice of sending broad and unspecified requests to more than one PIU was highlighted together with the need of the PIUs to check the data and information collected against databases ahead of sending a request to another PIU. In addition to that, the respondents to the survey indicated that not all PIUs are using the agreed PIU-PIU template to request data, which has been used by the majority of Member States in an effort to facilitate the exchange of PNR data (ibid.).

### Collection of data

The PIUs are becoming more and more operational and some, being in a more advanced position than others, mentioned that they often lack the necessary technological infrastructure to efficiently handle the vast amount of PNR data generated by air travel. The volume of data generated can overwhelm outdated systems, leading to delays and inefficiencies in processing critical information. PIUs may lack access to advanced technologies that facilitate large-scale data analysis. Without access to tools such as machine learning algorithms and big data analytics platforms, PIUs struggle to effectively process and analyse the vast amount of PNR data at their disposal.

Moreover, the acquisition of PNR data from travel agencies presents significant challenges attributable to a combination of logistical hurdles and several legal complexities (as explained under the section “legal matters”). Non-carrier economic entities, notably travel agencies and tour operators, may frequently deviate from the standardised data formats typically adhered to by air carriers. Consequently, this variance often culminates in incomplete datasets, thereby impeding the thoroughness of threat assessments.

According to Article 6 par. 2 of the Directive during the passengers’ assessment before their arrival or departure (a), The PIUs have the mandate to compare PNR data against databases on the grounds of terrorism and serious crime prevention, detection, investigation, and prosecution, always in line with existing EU, national, and international rules ( The European Parliament & the Council of the European Union, 2016a). However, from the replies received, the PIUs mentioned that they often face difficulties in establishing efficient connectivity with other databases, limiting their ability to cross-reference PNR data with other relevant information sources. This lack of integration hampers the effectiveness of security measures and compromises the ability to detect potential threats.

Lastly, the establishment of a reliable link between new and historical flights of the same individual poses a significant challenge for PIUs. Without efficient mechanisms in place to track individuals across different flights, identifying patterns of suspicious behaviour becomes increasingly difficult.

### Data quality

Another major challenge the PIUs are facing is that of the poor data quality. The quality of the data collected by the PIUs refers to representativeness, completeness, accuracy, and consistency of the data. The PIUs have to deal with the poor quality of data for various reasons. Usually, the passengers book their airline tickets on their own or through a travel agency. The respondents to the survey indicated regular ‘misspellings’ as an important issue which affects the data accuracy and completeness. In most cases misspellings are not done intentionally by the passengers or the travel agents, in order to hide a criminal action ( Glouftsios & Leese (2023)).

Regarding the completeness of data, the optional nature of certain passenger data also impacts data quality. It should be clarified that airline companies are not legally bοund to validate the data they are collecting. ΑΝΝΕΧ I of the Directive lists the data that, if collected by an air carrier in the normal course of their business, they should be transmitted to the PIUs, data like the dates of travel and travel itinerary, ticket information, contact details, travel agent, payment information, seat number, and baggage information are collected ( European Commission, n.d.). Based on the findings of the survey and review report on the implementation of the Directive ( European Commission, 2020a), it has been underscored that certain data categories must be mandated for collection by air carriers, with date of birth being deemed the most important. Furthermore, in addition to non-mandatory data collected, the absence of validation procedures prior to their acquisition from PIUs introduces inaccuracies. For instance, it is prevalent for travel agents to provide their contact details instead of those of the passengers during ticket booking. The responses received revealed that the PIUs face significant challenges related to both data quality and data processing limitations. On the data quality side, common issues include inaccuracies like misspellings and missing information, undermine the completeness and accuracy of the datasets available to PIUs. On the data processing side, the challenges occur due to the fact that there are no automated technological solutions that validate the quality of the data received. This results to manual verification, which ass extra workload on the PIU personnel and might cause delays in operational workflows. While these processing challenges do not directly impact the quality of the data itself, they do make it much harder for PIUs to manage and make use of PNR data effectively

To face the challenge of poor data quality, several PIUs cross-validate PNR data with data and information from other sources, most notable the API data, when applicable ( Glouftsios & Leese, 2023). Even though, the API data are considered more accurate, their collection is not mandatory for intra-Schengen flights, and the fact that ID’s checks are not always conducted at the airports, diminish the protentional of leveraging from the cross match between API and PNR data. Against this background, the possible adoption of the EU Regulation ( European Commission, 2022) 2019/818, is anticipated to address several data quality issues encountered by the PIUs and thus minimise the security gaps that they are currently facing with.

### Data analysis

Another factor that impacts both the quality and the subsequent analysis of the data collected is that of different PNRs for the same trip. Even though the Directive includes the ‘split/divided PNR information’ in the PNR data, it does not include the ‘linked PNR information’. The ‘split/divided PNR information’ refers to the process of dividing a PNR record locator into two or more, so as to handle the reservation separately, while the linked PNR is the process of connecting a PNR locator with another PNR locator. The latter is usually conducted upon request from the passenger or the travel agent in order for the passengers to sit together in the plane. Apart from that, it is possible for travel agents and passengers that are flying together to book separate tickets and not link them at all. While seemingly innocuous, this practice may obscure vital information from PIUs.

The capabilities of the PIUs are also affected by the practice of ‘broken travels’. Broken travels include multi-stage routes that combine many different modes of transport and is a technique highly preferred by terrorists for entering EU ( Hornak, 2015). Currently, the EU PNR Directive only covers the collection and processing of passenger data from air carriers, which limits PIUs' ability to get a full pictures of cross-border movements, in particular when other modes of transport are used. This gap is a limitation of the Directive itself, as it does not currently apply to other modes of transport. PIUs echoed this concern in their survey responses, highlighting that while the current framework is effective for air travel intelligence, it leaves significant gaps when individuals use disconnected legs of travel to potentially hide their movements. PIUs noted that without access to data from other modes of transport, it becomes much harder to spot suspicious travel patterns. Addressing this gap would mean expanding the Directive to include multi-modal travel data collection and integration, ensuring a more robust and holistic approach to travel intelligence.

### Staff matters

Since the adoption of the Directive ( The European Parliament and the Council of the European Union, 2016a), there has been a notable increase in the operational functionality of EU PIUs leading to one significant challenge, which is the efficient management of the vast amount of PNR data generated by air travel. The operational efficiency of PIUs is influenced by various factors, such as technological advancements and staff training. While some units have made significant strides in these areas, others lag behind due to resource constraints or organisational limitations. At the same time, several PIUs highlighted the need for trainings in data analysis, which is crucial for extracting insights from PNR data in order to also develop robust targeting rules. Without proper training, staff may struggle to effectively utilise available resources, leading to gaps in security.

### Legal matters

Lack of reciprocity with third countries further complicates efforts to effectively process PNR data. As highlighted in the Staff Working Document on Review of the Directive ( European Commission, 2020b), the intensification of requests for PNR data from third countries, not having a respective PNR Agreement with the EU, has, in some cases, led to retaliatory measures. For instance, reports indicate that certain third countries have directed their national airlines to cease PNR data transfers to the EU Member States, thereby affecting the implementation of the Directive and creating potential security risks. Additionally, air carriers may face conflicts of laws between EU data protection regulations and requirements from destination countries, potentially resulting in sanctions. It was thus considered imperative, from several PIUs, to introduce a comprehensive international cooperation strategy to facilitate the transfer of PNR data to and from EU countries, fully respecting fundamental rights.

In their survey responses, PIUs highlighted that they are facing challenged in adapting their operations to the evolving legal requirements - especially following the CJEU’s ruling in Case C-817/19 ( Court of Justice of the European Union, 2022). This judgement reinforced the need for strict necessity and proportionality when retaining PNR data. In practice, this means that PNR data can only be retained beyond the initial six months, if there is clear, objective evidence of a real risk linked to terrorism and serious crime. Moreover, the judgement makes a distinction between intra and extra EU flights requiring Member States to provide justification on the collection of PNR data for intra EU flights, with clear evidence of necessity and proportionality. PIUs have highlighted that this distinction poses additional operational challenges, as it necessitates a careful balancing of public security needs with fundamental rights, including privacy and data protection.

Last, the judgment also emphasised the need for meaningful human oversight in the use of automated systems for PNR data processing, however, PIUs have raised concerns about how transparent and reliable these systems are, stressing the need for clear guidelines to align with these legal standards. In addition, recent EDPS opinions on PNR agreements with countries like Canada ( EDPS, 2024), Norway ( EDPS, 2023a), Iceland ( EDPS, 2023b), and Switzerland ( EDPS, 2023c) have provided guidance, particularly on compliance with EU standards for data retention, oversight, and proportionality. While these opinions offer a framework for international data-sharing obligations, they also highlight the ongoing complexities faced by PIUs in meeting both operational and legal requirements ^7^.

### Recommendations

Although it is the PIUs that have mainly to deal with the above-mentioned challenges, their resolution requires a collaborative effort amongst policymakers, security practitioners, and industry to develop and promote innovation. Notably, technological advancements can enhance the operational capabilities of the PIUs, but their deployment is not sufficient to solve core problems. Based on the results of the survey, the present section presents a series of recommendations to fill the capability gaps of the PIUs that pertain to the technological, political, legal sphere. These suggestions are presented as initial steps that aim to enhance PIUs operations based on the challenges identified. Although the current article does not include a comprehensive analysis of the legal and operational implications their implementation may bring, future research could step on them to further explore the various aspects of their adoption.

### Validation of the data quality prior to their transmission to the PIUs

As previously described, the air carriers are not responsible for the validation of the data provided by the passengers. This implies that the passengers may provide information that is not verified until their collection from the PIUs, causing informational gaps and operational obstacles. An effective validation mechanism could enhance the data quality without causing additional cost to the air carriers. This mechanism shall have at its core tools that automatically validate the quality of the data and in case of mistakes, misspellings, or missing data do not allow the passenger to move to the booking of an airline ticket. In combination with the automatic verification of the contact details provided in the ticket reservation, this mechanism could significantly enhance the PIUs capabilities. The new provisional agreement on air passenger data moves towards this direction as establishes the automated data-collection and verification mechanisms to guarantee the accuracy of the data ( The Council of the European Union, 2024).

### Automation of data quality controls

Contrary to the previous recommendation which refers to the quality control prior their transmission to the PIUs, this recommendation refers to the validation of the data quality upon their collection by the PIUs. Some survey participants mentioned some tools that conduct data quality controls, but they are insufficient. This lack of automated data quality controls to check for possible errors, misspellings, invalid data poses a huge workload to the PIUs which they have to check manually the data transmitted by the air carriers. The development and deployment of tools that can automatically validate the data quality and detect mistakes or fields that have not been filled correctly could minimise the heavy workload of the under-staffed PIUs. In a subsequent step, the detected errors could be used to generate error reports per air carrier so as to inform the latter respectively.

### The development of automated tools that will generate rule suggestions based on previous hits

A recommendation stemming from the development of automated tools generating rule suggestions based on previous hits is to prioritise the refinement and enhancement of these automated systems. By leveraging advanced machine learning algorithms and data analytics, these tools can analyse historical hits or encounters, as well as feedback and validation from the PIU’s operators, thereby generating more accurate and effective targeting rule suggestions over time. The establishment of mechanisms for real-time feedback and validation from the operators will be essential to ensure the relevance and reliability of the generated rule suggestions. Those suggestions, that will include detailed explanations on the utilized criteria and relevant weights, could then be manually reviewed and potentially fine-tuned by the PIU experts and analysts and transformed into actual rules, that could be activated upon successful evaluation for a given time period. This process of refinement will not only enhance the efficiency and effectiveness of targeting efforts but also enable the adaptation to evolving threats and changing operational environments.

### Standardisation of data transfers to third countries and to Competent Authorities (CA)

In order to enhance data transfers between PIUs and third countries, several steps can be taken. Firstly, leveraging EU Security Research initiatives is suggested as effective starting points for involving PIUs and standardizing data sharing practices. However, it is emphasized that additional funding is imperative to sustain and expand such endeavours. Secondly, there is a consensus among multiple PIUs on the necessity to establish uniform and secure channels for data exchange, particularly with third countries and NCAs. While some PIUs currently rely on email for data transfer, many advocate for the implementation of automated solutions to streamline this process. Thirdly, it has been proposed to revise existing data exchange methods, such as the excel template utilised for sharing passengers' data via Europol’s SIENA message system ( Europol, 2022). Suggestions include incorporating drop-down menus to minimize errors and enhance efficiency in data transmission. These recommendations aim to facilitate smoother and more secure data transfers, thereby improving collaboration and information exchange among relevant stakeholders.

### Standardisation of transfers of data from non-carrier economic operators

The operational value of the collection of the information from non-carrier economic operators, like tour operators and travel agencies was highlighted in the review of the implementation of the Directive ( European Commission, 2020a). A recommendation for the standardisation of transfers of data from non-carrier economic operators is to establish clear and uniform protocols and guidelines for data transfer processes. This entails developing standardised formats, protocols, and encryption methods to ensure secure and efficient transmission of data between non-carrier economic operators and PIUs. Additionally, providing comprehensive training and support to non-carrier economic operators on data protection regulations, privacy safeguards, and compliance requirements will be crucial to ensure adherence to established standards, EU, and national legislation. By promoting consistency and coherence in data transfer procedures, standardization efforts can enhance interoperability, streamline operations, and mitigate potential risks associated with data breaches or inconsistencies, ultimately contributing to more effective and harmonized security measures across borders.

### The collection of specific passenger’s data elements shall become mandatory

In light of the current voluntary nature of the collection of PNR data as outlined in Annex I of the Directive and considering that air carriers are expected to transfer data collected in the normal course of their business, exists a potential challenge regarding data availability. To address this, it is recommended that certain key data elements are made mandatory for collection by air carriers. These mandatory data fields should include but not be limited to: date of birth, contact details, API, payment method, date of booking, final confirmation on the boarding status of passengers indicating whether they have boarded or not, itinerary details, information regarding travel companions when applicable, and document information, including number, issuance country, expiration date, and citizenship. Ensuring the mandatory collection of these data elements would enable analysts to have a comprehensive dataset necessary for conducting thorough assessments. This recommendation is crucial for enhancing the overall quality and completeness of the data available for analysis and decision-making within the aviation security domain.

### Expansion of the PIUs’ mandate to other modes of transport

It is recommended that the European Union and its Member States adopt a unified and coordinated approach towards the expansion of travel data collection beyond air carriers to include other modes of transport such as maritime, trains and buses, in full compliance with the principle of proportionality and necessity. This holistic approach is essential for enhancing cross-border cooperation in combating terrorism and serious crime, as it ensures security measures cover multiple transportation modes, reducing the potential for individuals to exploit gaps in information sharing. By extending collaboration among PIUs to encompass various modes of transport, security analysts can obtain a more comprehensive view of a traveller's journey, thereby minimising operational gaps posed by "broken travels" and indirect routes. Additionally, this unified approach streamlines efforts, reduces duplication of resources, and fosters public confidence in passenger safety and security. Towards this direction, ( The Council of the European Union, 2019) to explore the necessity and feasibility of collecting, storing, and processing of PNR data from cross-border forms of transport other than air traffic the Council of the European Union, is moving towards the right direction has already made recommendations, indicating progress in the right direction.

## Conclusions

The survey conducted in the context of the TENACITy project showed the diverse nature of the challenges that accompany all the phases of the PNR data processing, from the transfer phase to the collection, and process phase. The present study focused on the operational effectiveness of PIUs, taking a closer look at the broader goals of the PNR Directive would be a worthwhile direction for future research. The lack of commonly used standards by all the involved stakeholders and the poor data quality can be placed at the base of the challenges faced by the PIUs that cause a ‘domino effect’ to their operational capabilities. Consequently, there is a collective desire for certain data elements to be mandated for collection by air carriers, such as passengers' dates of birth. It is noteworthy though that the nature of the challenge may differ from the nature of a possible solution. For example, most of the PIUs participated to the survey stated that the existing technological tools cover a big part of their operational needs, yet a possible solution to the lack of personnel to deal with the heavy workload, could be the development of automated tools to perform quality controls to the PNR data received. Moreover, while some PIUs are currently processing PNR data from carriers lacking automated transmission capabilities, the availability of datasets for comparison remains limited. This challenge extends to PIUs collecting PNR data from other modes of transport.

The Directive has marked air travel intelligence in the EU and put the PIUs in the centre of it. Its review showed the significant added value of their creation and operation in the field of travel intelligence, and crime and terrorist prevention. Therefore, it is important to explore the ways on how they can be evolved in the future and by extension to further support travel intelligence strategies. The identification of key challenges confronting PIUs serves as an initial step in this trajectory, paving the way for informed enhancements and ensuring the continued efficacy of travel intelligence initiatives in full respect of fundamental rights.

## Consent to participate

All participants provided informed consent by filling an online information sheet and consent form for participation in the survey conducted as part of the TENACITy project.

## Data Availability

The sensitive nature of the TENACITy project necessitates strict restrictions on the disclosure of survey data. Making the survey data openly available poses significant security and privacy risks, as it could potentially compromise the confidentiality of respondents and reveal sensitive operational details of Passenger Information Units. Access to anonymised survey data will be granted on a case-by-case basis, subject to the approval of the Security Advisory Board of the TENACITy project. Interested readers and reviewers may request access by contacting the authors via email. Zenodo: TENACITy, Navigating the challenges of passenger name record data and the way forward DOI: https://doi.org/10.5281/zenodo.12743146 Aposkiti & Makri (2024). This project contains the following underlying data: Questionnaire Creative Commons Attribution 4.0 International: https://creativecommons.org/licenses/by/4.0/

## Footnotes

^1^ More information on the Entry Exist system can be accessed via: https://travel-europe.europa.eu/ees_en.

^2^ More information on ETIAS can be accessed via https://travel-europe.europa.eu/etias_en.

^3^ As mentioned in recital no.9 of the Directive (2016/681), *some air carriers retain as part of the PNR data the API data they collect, while others do not. The use of PNR data together with API data has added value in assisting Member States in verifying the identity of an individual, thus reinforcing the law enforcement value of that result, and minimising the risk of carrying out checks and investigations on innocent people. It is therefore important to ensure that where air carriers collect API data, they transfer it irrespective of whether they retain API data by different technical means as for other PNR data.* It is important to underscore that, for the purposes of the present Article, any reference to PNR data presupposes adherence to the definitional parameters outlined within the Directive.

^4^ More information about the TENACITy project can be accessed via: https://tenacity-project.eu/

^5^ Due to confidentiality considerations and the discretion required regarding the origins of responses, the authors have opted not to disclose whether the replies received originate from European Union Passenger Information Units (PIUs) or from third countries.

^6^ More information are available here: https://www.wcoomd.org/en/topics/facilitation/instrument-and-tools/tools/api-pnr.aspx?p=1

^7^ For further insights into the legal aspects of PNR data regulation, see Vogiatzoglou *et al*. (2020), Thönnes & Vavoula (2023), and Gerards (2023).

## References

[ref-1] Aposkiti C Makri F : Navigating the challenges of passenger name record data and the way forward. *Zenodo.* 2024. 10.5281/zenodo.12743231 PMC12395126 40894387

[ref-2] Court of Justice of the European Union: CJEU Press Release No 105/22: (21 June 2022). The Court considers that respect for fundamental rights requires that the powers provided for by the PNR Directive be limited to what is strictly necessary. Judgment of the Court in Case C-817/19 | Ligue des droits humains. Court of Justice of the European Union, Luxemburg,2022. Reference Source

[ref-3] De Hert P Papakonstantinou V : The EU PNR framework decision proposal: towards completion of the PNR processing scene in Europe. *Comput Law Secur Rev.* 2010;26(4):368–376. 10.1016/j.clsr.2010.05.008

[ref-4] European Commission: Commission Implementing Decision (EU) 2017/759 of 28 April 2017 on the common protocols and data formats to be used by air carriers when transferring PNR data to Passenger Information Units C/2017/2743 OJ L 113, 29.4.2017. Official Journal of the European Union,2017;48–51. Reference Source

[ref-5] European Commission: 128 final Report from the Commission to the European Parliament and the Council on the review of Directive 2016/681 on the use of Passenger Name Record (PNR) data for the prevention, detection, investigation and prosecution of terrorist offences and serious crime {SWD (2020) 128 final}. Brussels,2020a. Reference Source

[ref-6] European Commission: Passenger data. Migration and home affairs. (n.d.). Reference Source

[ref-7] European Commission: 128 final commission staff working document accompanying the document report from the commission to the european parliament and the council on the review of Directive 2016/681 on the use of Passenger Name Record (PNR) data for the prevention, detection, investigation and prosecution of terrorist offences and serious crime {COM (2020) 305 final}. Brussels,2020b. Reference Source

[ref-8] European Commission: Proposal for a regulation of the European Parliament and of the council on the collection and transfer of advance passenger information for the prevention, detection, investigation and prosecution of terrorist offences and serious crime, and amending Regulation (EU) 2019/818 COM(2022)731 final. Strasburg,2022. Reference Source

[ref-23] European Data Protection Supervisor: Opinion 15/2024 on the signing and conclusion of an Agreement between the EU and Canada on the transfer of Passenger Name Record (PNR) data.2024. Reference Source

[ref-24] European Data Protection Supervisor: Opinion 45/2023 on the negotiating mandate for an Agreement between the EU and Norway on the transfer of Passenger Name Record data.2023a. Reference Source

[ref-25] European Data Protection Supervisor: Opinion 46/2023 on the negotiating mandate for an Agreement between the EU and Iceland on the transfer of Passenger Name Record data.2023b. Reference Source

[ref-26] European Data Protection Supervisor: Opinion 47/2023 on the negotiating mandate for an Agreement between the EU and Switzerland on the transfer of Passenger Name Record data.2023c. Reference Source

[ref-9] Europol: Secure Information Exchange Network Application (SIENA): ensuring the secure exchange of sensitive and restricted information.2022. Reference Source

[ref-27] Gerards J : Machine learning and profiling in the PNR system. Verfassungsblog,2023. Reference Source

[ref-10] Glouftsios G Leese M : Epistemic fusion: passenger information units and the making of international security. *Rev Int Stud.* 2023;49(1):125–142. 10.1017/S0260210522000365

[ref-11] Hornak L : ‘Broken travel’ keep ISIS’ western recruits off the radar of intelligence agencies. The World,2015. Reference Source

[ref-12] International Air Transport Association: Passenger and airport data interchange standards xml implementation guide - PNRGOV (version 16.1).2016. Reference Source

[ref-13] Kanellopoulos AN : Travel intelligence as a tool for counterintelligence and border security. *Security and Defence Quarterly.* 2024;45(1):55–67. 10.35467/sdq/174523

[ref-14] Reja U Manfreda KL Hlebec V : Open-ended vs. close-ended questions in web questionnaires. *Developments in Applied Statistics.* 2003;159–177. Reference Source

[ref-15] Roopa S Rani M : Questionnaire design for a survey. *J Indian Orthod Soc.* 2012;46(4):273–277. 10.5005/jp-journals-10021-1104

[ref-16] The Council of the European Union: Council Directive 2004/82/EC of 29 April 2004 on the obligation of carriers to communicate passenger data OJ L 261, 6.8.2004. Official Journal of the European Union,2004;24–27. Reference Source

[ref-17] The Council of the European Union: Council conclusions on widening the scope of the use of Passenger Name Record (PNR) data to forms of transport other than air traffic. Council conclusions (2 December 2019), Brussels. 14746/19.2019. Reference Source

[ref-18] The Council of the European Union: Air passenger data: council and the European Parliament reach provisional agreement to increase security and enhance border management. [Press release].2024. Reference Source

[ref-19] The European Parliament and the Council of the European Union: Directive (EU) 2016/681 of the European Parliament and of the Council of 27 April 2016 on the use of Passenger Name Record (PNR) data for the prevention, detection, investigation and prosecution of terrorist offences and serious crime. Official Journal of the European Union. OJ L 119, 4.5.2016.2016a;132–149. Reference Source

[ref-20] The European Parliament and the Council of the European Union: Regulation (EU) 2016/399 of the European Parliament and of the Council of 9 March 2016 on a Union Code on the rules governing the movement of persons across borders (Schengen Borders Code) (codification). Official Journal of the European Union,2016b. Reference Source

[ref-28] Thönnes C Vavoula N : Automated predictive threat detection after Ligue des Droits Humains. Verfassungsblog: On Matters Constitutional,2023. Reference Source

[ref-21] US Government Publishing Office: Aviation and transportation security act: to improve aviation security and for other purposes. Washington, D.C. S.1447 - 107th US Congress,2001. Reference Source

[ref-29] Vogiatzoglou P Tavárez KQ Fantin S : From theory to practice: Exercising the right of access under the Law Enforcement and PNR Directives. JIPITEC,2020;11(3). Reference Source

[ref-22] World Customs Organisation: Challenges and opportunities of passenger data systems.2015. Reference Source

