# Supplementary material for: Navigating the challenges of passenger name record data and the way forward
Source: Open Res Eur. 2025 May 19;4:168. Originally published 2024 Aug 5. [Version 2] doi: 10.12688/openreseurope.18037.2 (PMC12395126; doi:10.12688/openreseurope.18037.2)
Supplement: Peer review history

# Reviewer report: Reviewer response for version 2

**DOI:** 10.21956/openreseurope.21857.r54576

**Published:** 2025-08-30

**Authors:** Asif Qumer Gill

**Affiliations:** 1 University of Technology Sydney, Ultimo, Sydney, Australia

Based on the review of the revised version and also the previous revision and comments. There are major flaws that may be fixed via just revision. 

 1. Sample size of 14 is not adequate of making any kind of substantial claims. This has been acknowledged in the limitations, however, this is major limitation and undermine the meaningful quality of the contribution. Research may consider conducting additional research. 

 2. Similarly, limitations acknowledge the lack of bias testing. However, given the details about the data and how was it interpreted are not clear, thus, it is extremely difficult to establish the research contribution. 

 3. There needs to be a separate section on "Research Buckhound and Related Work" before the research method section. This is important to justify the need for research via comparison with similar or related studies. 

 4. An appropriate research design model and a set of hypothesis can be defined using some theoretical lens.

If applicable, is the statistical analysis and its interpretation appropriate?

No

Is the study design appropriate and is the work technically sound?

Partly

Is the work clearly and accurately presented and does it engage with the current literature?

Partly

Are the conclusions drawn adequately supported by the results?

Partly

Are sufficient details of methods and analysis provided to allow replication by others?

Partly

Are all the source data and materials underlying the results available?

No

Reviewer Expertise:

Please see my earlier comments.

I confirm that I have read this submission and believe that I have an appropriate level of expertise to state that I do not consider it to be of an acceptable scientific standard, for reasons outlined above.

# Reviewer report: Reviewer response for version 2

**DOI:** 10.21956/openreseurope.21857.r54577

**Published:** 2025-08-29

**Authors:** Elaine Fahey

**Affiliations:** 1 The City Law School, City St. Georges, University of London, London, England, UK

The article is difficult to follow as to its aims and methods and employs a variety of headings that could be better explained. Although it was a deserving study to be undertaken for its public importance, the scientific presentation of the challenge to be studied and the outcomes of the empirical work were complex to understand. The disciplinary norms governing the analysis of the data were not clearly stated. It is difficult to see the legal research question to be studied. The study was a primarily empirical study of the use of PIU data. It was difficult to follow the thread of its logic and methods in the absence of any form of presentation of data intelligibly. The place of security institutes and the police force in the study was difficult to understand in the absence of significant ethical disclaimers that were not present despite its relative sensitivity. There was insufficient explanation of the elementary concept of the PIU, its work, its data sources and relationships between the units. The validation ‘point’ of the data needed clearer presentation.  The number of data units was important and more could be explained as to broader studies of such data, the units, their engagement eg in tables, graphs. The findings made drew attention to the question of the subjects and objects of the study which appeared to criticise the public overall and more work was needed on the framing of this finding. The use of term ‘stakeholder’ needed clarification which possibly needed more development throughout. Findings were expressed as results in a fashion that was difficult to comprehensive. The survey and its results could have been more clearly presented and explained. More was needed on the country of the PIUs responding and its relationship to the implementation of the PNR directive. There was limited engagement with EU institution reports on the implementation of the PNR directive.  A distinction between intra-EU and extra-EU data needed to be made. The paper appeared to emphasise more the extra-EU transfers and while eg important EDPS opinions were referenced. The piece was unduly descriptive overall despite reaching significant conclusions as to accountability and governance.  The relationship between EU law and international law needed to be developed briefly to explain more accurately the source of the obligations, which is only tentatively sketched. Some countries had chosen not to have PNR agreements with the EU and this perhaps might assist the inquiry although it is still not clear to be the precise research question to be addressed.

If applicable, is the statistical analysis and its interpretation appropriate?

I cannot comment. A qualified statistician is required.

Is the study design appropriate and is the work technically sound?

Partly

Is the work clearly and accurately presented and does it engage with the current literature?

Partly

Are the conclusions drawn adequately supported by the results?

Partly

Are sufficient details of methods and analysis provided to allow replication by others?

No

Are all the source data and materials underlying the results available?

No

Reviewer Expertise:

EU law, EU external relations, AFSJ, digital/ data governance

I confirm that I have read this submission and believe that I have an appropriate level of expertise to confirm that it is of an acceptable scientific standard, however I have significant reservations, as outlined above.

# Reviewer report: Reviewer response for version 2

**DOI:** 10.21956/openreseurope.21857.r54383

**Published:** 2025-08-28

**Authors:** Laura Drechsler

**Affiliations:** 1 Law, KU Leuven, Leuven, Flanders, Belgium

No further comments after the revision of the authors.

If applicable, is the statistical analysis and its interpretation appropriate?

Not applicable

Is the study design appropriate and is the work technically sound?

Yes

Is the work clearly and accurately presented and does it engage with the current literature?

Yes

Are the conclusions drawn adequately supported by the results?

Yes

Are sufficient details of methods and analysis provided to allow replication by others?

Partly

Are all the source data and materials underlying the results available?

No

Reviewer Expertise:

Data protection law, border management law, fundamental rights, EU law

I confirm that I have read this submission and believe that I have an appropriate level of expertise to confirm that it is of an acceptable scientific standard.

# Reviewer report: Reviewer response for version 2

**DOI:** 10.21956/openreseurope.21857.r54573

**Published:** 2025-06-18

**Authors:** Niovi Vavoula

**Affiliations:** 1 University of Luxembourg, Esch-sur-Alzette, Luxembourg

The authors have provided a revised version of their article, which partly addresses the comments but it is still not ready for indexing. In particular, the sections on methodology, legal matters and recommendations are not taking into account in full the relevant literature and the CJEU judgment in Ligue des Droits Humains. The analysis is oriented towards improving effectiveness from a PIUs perspective without balancing with other considerations such as fundamental rights and free movement.

 Please find below some detailed comments:

- The first sentence of the article is too complex, and I recommend dividing it in two. - 'More recently, these efforts include the Entry Exit System (EES) ^1^ and the European Travel Information and Authorisation System (ETIAS) which are not still in operation': These are not the only efforts in digitalised border management in recent years (e.g. Frontex and Eurosur as well as other IT systems).
- 'Advance Passenger Information (API) Directive' This Directive is now reformed, and it should be connected with PNR data. API is a sub-set of PNR data.
- 'In this context, the use of PNR data has been integral to the international cooperation efforts of the EU against terrorism and serious crime for nearly two decades with the implementation of the Passenger Name Records (PNR) Directive (EU) 2016/681 (henceforth “the Directive”) being the cornerstone of them.' This is a shortcut, considering that the EU first entered into agreements with specific third countries and then looked into an EU PNR system. - 'transmission, collection, and processing of PNR data': by whom to whom?
- The authors could be more specific as to which aspects in the PNR data processing they refer.  - What is the precise research question of the publication? - 'all PIUs participating in the TENACITy consortium and the EU PIUs': could the authors be more specific? How many PIUs where contacted? How many of these were not EU PIUs?
- 'To start with, despite the sample size offering a diverse mix of roles and functions, it can be considered as relatively small (14 PIUs).' how many PIUs were contacted? How? How much period was given for answers? What questions where answered? Yes or no, or for further development? Was there a follow-up as regards the authorities that did not respond? Did they deny participation? - The word 'custodians' is unclear: do the authors mean data controllers? Do they mean something different?
- Τhe authors state that the lack of non-standard format creates operational gaps. Could they offer some examples? Are data missing? - Is the lack of spontaneous transfers such a problem when there are so many requests for data transfers by PIUs?
- What is the solution to the increased requests from PIU-to-PIU transfer? To have specific conditions and rules to abide by? Is the increased number of requests disproportionate? - On the section on data quality there is some repetition that could be ironed out.
- ‘Possible adoption of the EU Regulation ( European Commission, 2022) 2019/818’: this needs update. - On data analysis: what are the challenges on expanding PR collection/analysis to other modes of transport? The authors mention what the PIUs stated and seem to uncritically accept it, but it would be useful to have any counter-arguments e.g. wouldn’t this tremendously increase the work of PIUs which already struggle? Would it affect free movement? E.g. such discussions have emerged in the context of the API proposal.
- When it comes to the legal matters the authors refer to the concerns raised by PIS on how transparent and reliable automated systems are. However, this is a major concern that needs to be mentioned earlier potentially on data analysis. Here it is only mentioned in relation to oversight, which is not really dealt with. What are the specific concerns with oversight? How should it take place? Later it is mentioned as a recommendation but again without engaging to potential biases/non-discrimination concerns.
- There is limited engagement with the judgment of the CJEU in Ligue des Droits Humains and the recommendations do not take the judgment in account.
- The methodology could be further clarified.

If applicable, is the statistical analysis and its interpretation appropriate?

Partly

Is the study design appropriate and is the work technically sound?

Partly

Is the work clearly and accurately presented and does it engage with the current literature?

Partly

Are the conclusions drawn adequately supported by the results?

Partly

Are sufficient details of methods and analysis provided to allow replication by others?

Partly

Are all the source data and materials underlying the results available?

Partly

Reviewer Expertise:

EU Area of Freedom, Security and Justice and Cybersecurity

I confirm that I have read this submission and believe that I have an appropriate level of expertise to confirm that it is of an acceptable scientific standard, however I have significant reservations, as outlined above.

# Reviewer report: Reviewer response for version 1

**DOI:** 10.21956/openreseurope.19492.r42906

**Published:** 2024-09-16

**Authors:** Maria Tzanou

**Affiliations:** 1 The University of Sheffield, Sheffield, England, UK

Summary of the article

 The article explores the challenges faced by Passenger Information Units (PIUs) regarding the collection, handling and use of Passenger Name Record (PNR) data following the implementation of the EU PNR Directive (EU) 2016/681. According to the authors,  it employs a combination of research and survey methodologies to gather data from PIUs.

 The study identifies two main obstacles faced by (PIUs): the absence of standardised practices and issues of data quality. It is also argued that fragmented implementation and regulatory barriers are important hindrances to the optimal utilisation of PNR data for counterterrorism and crime prevention efforts.

 To address these challenges, the article proposes that technological tools, in particular automated processing could present a solution.

 Review

 Overall, the article makes a valuable contribution to ongoing debates on EU travel intelligence. In particular, the empirical research provides useful insights to the operational aspects of PIUs.

 The analysis would benefit from several suggestions that I discuss below. These comprise both analytical and methodological aspects of the discussion:

 1. As the authors themselves admit, the methodology employed has some limitations. The most important of these are not explicitly acknowledged in the study nor any discussion is provided on how these could be mitigated or addressed. It is reported that ‘Fourteen PIUs replied to the survey’ and ‘due to confidentiality considerations and the discretion required regarding the origins of responses, the authors have opted not to disclose whether the replies received originate from European Union Passenger Information Units (PIUs) or from third countries’.

 However, this makes it very difficult to assess whether the results reported are indeed robust and whether they represent the views of PIUs’ across all the EU Member States. 14 responses appear limited for this type of research and the authors should have explained more clearly the representativeness of the sample and the results. Ideally, this would go further than stating that ‘the survey results broadly align with the Commission’s report. This is because it less clear what the contribution of this research/ survey has added to the Commission’s findings.

 2. While the confidentiality considerations and concerns are valid, the article would have made a better contribution if it chose to present some aggregated, anonymised, statistical data which support the arguments made therein. If this is not possible, the article could have at least included in the appendix the survey questions used to ensure that reviewers and readers could assess these.

  3. It is argued that ‘Inclusion criteria for the review encompass research studies, reports, and evaluations specifically focused on the implementation of the PNR Directive and Passenger Information Units (PIUs), incorporating both qualitative and quantitative methodologies such as surveys, interviews, case studies, and observational studies. Systematic reviews and meta-analyses that provide data on PIUs are also included.’ And that ‘Additionally, studies with significant methodological flaws or a high risk of bias, those not involving users or stakeholders of PIUs …are excluded.’

 It is not sufficiently clear where the included sources are provided (there is no further reference to these) and what is their purpose. The article could have provided a comprehensive review of the literature on the EU PNR Directive (including the relevant CJEU judgment, which is very relevant to these discussions). At the same time, it is not clear which sources were excluded and why. What studies have ‘significant methodological flaws’, who decided on this and on the basis of what criteria?

 4. The full background regarding the adoption of the PNR Directive at the EU level could have been discussed in more depth. Instead the article does not mention anything about this context and only focuses on the 9/11 attacks and relevant measures. It seems, therefore to be missing out on particular ‘European realities and needs’ in this context. More importantly, it is not clear if these issues were surveyed among PIUs, for instance, do PIUs’ related actors consider that the EU PNR Directive is achieving its objectives? The discussion would have benefitted significantly if it offered an analysis of these issues.

 5. The discussion of the results of the survey could have been clearer. Below I discuss some cases/ examples which I consider important:

 a) It is argued that ‘the PIUs are facing data standardisation issues. First of all, it should not be taken for granted that all the actors involved in the PNR/API data transfer implement existing standards.’

 It would be useful if the article explained further what these standardisation issues entail and which are not implemented. Would this be considered a case of non-compliance with the EU PNR Directive or not? There is no clear analysis provided on this.

 b) Furthermore, it is mentioned that ‘according to the survey, some air carriers may not support automated transmissions of PNR data, making it challenging for PIUs to collect comprehensive datasets. This lack of data from air carriers undermines the effectiveness of security measures and compromises the ability to identify unknown threats.’ However, the fact that ‘some air carriers do not support automated transmissions’ does not mean that there is ‘a lack of data’. This point should be clarified as it causes confusion regarding the exact nature of the issues identified by the survey.

 c) It is argued that ‘Yet, based on the answers received, even the data quality check conducted by the PIUs is challenging for them, as it has to be performed manually. The absence of automated technological solutions to monitor the validity and quality of the data received causes a huge workload for the PIU personnel.’

 This discussion is confusing as it confounds data quality issues with processing issues/ limitations of the data faced by PIUs. The latter concern the processing/ handling of the data and not data quality itself under which it is discussed.

 d) It is argued that ‘The capabilities of the PIUs are also affected by the practice of ‘broken travels’. Broken travels include multi-stage routes that combine many different modes of transport and is a technique highly preferred by terrorists for entering EU (Hornak, 2015). By collecting and handling passengers’ data only from air carriers, the PIUs can have only a limited intelligence picture of the cross-border movements, which constitutes an important security gap.’

 It is unclear whether this is a limitation of the EU Directive itself or whether it is an issue of implementation. In any case, the article would benefit by providing evidence supported by the survey responses on these matters.

 6. The proposed recommendations could have been more expanded and comprehensive- around one paragraph for each identified does not seem to provide sufficient depth or breadth to the discussion. The development of automated tools is proposed as a recommendation for many of the issues identified without any further discussion of the capabilities, limitations and potential risks of such mechanisms (including those identified by the CJEU).

If applicable, is the statistical analysis and its interpretation appropriate?

Not applicable

Is the study design appropriate and is the work technically sound?

Partly

Is the work clearly and accurately presented and does it engage with the current literature?

Partly

Are the conclusions drawn adequately supported by the results?

Yes

Are sufficient details of methods and analysis provided to allow replication by others?

No

Are all the source data and materials underlying the results available?

No

Reviewer Expertise:

data protection, privacy, fundamental rights, EU Law

I confirm that I have read this submission and believe that I have an appropriate level of expertise to confirm that it is of an acceptable scientific standard, however I have significant reservations, as outlined above.

# Response

**Published:** 2025-04-28

**Authors:** Christiana Aposkiti

Thank you for your feedback. 

1. In response to your comments regarding the limitations of the methodology, we have revised the article to more explicitly acknowledge the constraints of our research, particularly regarding the sample size and the anonymous nature of the survey. We now provide a detailed explanation of the representativeness of the sample, highlighting the diverse roles of the respondents and how this diversity helps mitigate the impact of the limited number of responses. We have also added suggestions for future research, including the potential to use interviews or workshops to gather more in-depth data and increase participation. 

1. Thank you. Indeed, the questionnaire is included under the “Extended data” sub chapter. 

1. The focus of our article is on presenting the findings from a survey conducted with EU Passenger Information Units (PIUs) as part of the TENACITy project. Our primary methodology involved direct stakeholder input via surveys, rather than a comprehensive systematic literature review. To address your comment we clarified in the text that no specific studies were excluded from our analysis based on methodological flaws or risk of bias. Instead, our inclusion criteria primarily focused on the direct relevance of studies and reports to the implementation of the PNR Directive and the operations of PIUs, incorporating both qualitative and quantitative methodologies. 

1. We acknowledge the importance of discussing the broader context of the PNR Directive’s adoption and its alignment with European realities and needs. However, our research was intentionally scoped to focus on the operational effectiveness of the Directive, specifically addressing challenges faced by Passenger Information Units (PIUs) in their daily activities, particularly concerning technological and procedural aspects. A detailed examination of the historical and political motivations behind the Directive’s adoption, as well as its overarching policy objectives, fell outside the scope of this study. That said, a few additions on EU historical context have been added. In addition, we did provide PIUs with the opportunity to highlight challenges beyond the technological and procedural domains, and these findings have been included in the article. While some PIUs commented on operational gaps related to the Directive’s implementation, their responses primarily reflected practical challenges rather than evaluations of the Directive’s broader objectives. We will clarify this point in the text to ensure that the study’s scope and its focus on operational issues are clearly communicated to readers. 

A) In the article we specifically highlight that: Some air carriers may not support automated transmissions of PNR data, making it challenging for PIUs to collect comprehensive datasets. This undermines the effectiveness of security measures and hampers the ability to identify unknown threats. The EDIFACT PNRGOV standard is a message standard, not a content standard, leading to potential variations in data formats or duplications, complicating data management. These examples illustrate the operational challenges stemming from inconsistent implementation of standards. While these issues do not necessarily constitute non-compliance with the EU PNR Directive, they expose gaps in the uniform application of the standards necessary for optimal functioning. We will ensure this explanation is further emphasised to enhance clarity for readers. B) C) D): relevant sections have been adjusted.  

1. The development of automated tools was emphasised as a key recommendation because it directly addresses the operational challenges identified by PIUs, such as the heavy manual workload and issues with data quality validation. While further discussion of the technological and legal aspects would be valuable, this remains a potential area for future research rather than the focus of the current study. To clarify our intent and improve transparency, we will revise the recommendations section to better articulate that these suggestions are intended as initial, practical steps to enhance PIU operations, rather than exhaustive analyses of their broader implications. 

# Reviewer report: Reviewer response for version 1

**DOI:** 10.21956/openreseurope.19492.r42901

**Published:** 2024-09-11

**Authors:** Elif Mendos Kuşkonmaz

**Affiliations:** 1 University of Essex, Colchester, England, UK

**General Comment**

 The work promises to contribute to the research conducted on the practices of PIUs. Generally, the literature from legal and other disciplines on PNR schemes thus far engages with theoretical questions (Amoore, 2009 [Ref-1]; Mitsilegas, 2010 [Ref-4]) or the inquiries around the implementation of the EU PNR Directive in certain jurisdictions (Irion et al., 2021 [Ref-3];  Olsen and Wiesener, 2021 [Ref-5]). As far as I am aware, one article published in English covers the practices of several EU PIUs carried out by Glouftsikos and Leese, to which the authors refer.

 In light of this, the authors raise a valid question focusing on the PIUs and employ a suitable method (e.g., a survey) to answer that question. They are explicit about the limits of the chosen method. The work follows a logical structure and partly relies on sources to substantiate the points raised. In the end, the authors make several recommendations to inform PNR schemes based on the survey results.

 Although the survey results and recommendations promise to instigate policy change, the work might need further development in light of the review questions. Below, I made recommendations based on questions 1, 3, and 6.  

**Q1: “Is the work clearly and accurately presented and does it engage with the current literature?”**

 I answered this question ‘partly’ because I think the authors could enrich their explanation of the place of the work against the existing literature with more specific examples. The authors express that “[T]he document seeks to contribute to the existing literature concerning travel intelligence within the European Union, which currently remains somewhat limited in scope and depth”. In addition to this brief statement and a few academic references, specifically on PNR schemes, the work could engage with the literature more comprehensively by citing the literature, what it covers and why the perspective of the existing works is limited to a few words. The authors cite the recent work by Glouftsikos and Leese and the 2010 article questioning the legality of the EU PNR Directive by de Hert and Papakonstantinou. Still, they do not directly consider where their work fits within the existing literature. Being more explicit in terms of the existing gap in the literature and the work’s contribution to fill that gap with further references to the existing works could strengthen its engagement with the literature further.

**Q2: “** **Are sufficient details of methods and analysis provided to allow replication by others?”**

 I answered this question ‘partly’ because I think the methods section could be revised to bring more precision. Under this section, the authors mention that “[i]nclusion criteria for the review encompass research studies, reports, and evaluations specifically focused on the implementation of the PNR Directive and Passenger Information Units (PIUs),..”. The work could benefit from explaining more clearly the review the authors conducted as a method because it is not directly clear what this review refers to, given that the work is overall based on a survey.

**Q6: “** **Are the conclusions drawn adequately supported by the results?”**

 I answered this question ‘partly’ because the recommendations do not sufficiently address the legal matters arising from the survey results. This is clear when the recommendations do not refer to the PIUs’ concerns over data retention. Also, some recommendations overlook the general issues arising from the existing (and also pending) CJEU case law.

 In terms of conclusions, the authors provide several recommendations regarding the survey results. As the authors mention, the recommendations generally encompass technological solutions. I want to highlight that so-called technological issues can have direct or indirect legal consequences under EU law. On the one hand, I understand that the objective of the work needs to cover addressing legal issues arising from PNR processing under EU law. On the other hand, the authors recognise that Based on the survey results, the present section presents a series of recommendations to fill the capability gaps of the PIUs that pertain to the technological, political, *legal sphere*” [emphasis added]. There is, however, little recognition of the legal limits and standards of what the PIUs can do. For example, the authors recommend the development of automated tools and refer specifically to machine learning algorithms [“By leveraging advanced machine learning algorithms and data analytics, these tools can analyse historical hits or encounters, as well as feedback and validation from the PIU’s operators, thereby generating more accurate and effective targeting rule suggestions over time”]. I would like to note here that following the CJEU’s *Le Ligue des droits humains* decision, which the authors recognise under the heading on legal matters, there is a discussion among the legal scholars about whether the CJEU’s analysis of machine learning algorithms could be interpreted as a prohibition of those algorithms where they are detrimental to individuals right to effective remedy (See: CJEU, *Ligue des droits humains*, para. 195. See also: Rotenberg, 2022 [Ref-6]). Moreover, the recommendation of making certain passenger data mandatory does not consider what this would mean their collection would come under the Law Enforcement Directive instead of the GDPR (CJEU, *Ligue des droits humains*, para. 81). Finally, the recommendation on expanding the PIUs’ mandate to other modes of transfer does not indicate if this would include intra-EU routes but assuming that the authors consider those routes as well, the legal implications of this expansion – which the CJEU touched upon in *Ligue des droits humains*, are left out (Brouwer, 2023 [Ref-2]). This oversight does not address the fundamental rights protection afforded to individuals with respect to the processing of PNR data – a point that the authors make an effort to recognise (“The identification of key challenges confronting PIUs serves as an initial step in this trajectory, paving the way for informed enhancements and ensuring the continued efficacy of travel intelligence initiatives *in full respect of fundamental rights*”), but do not at the end consider in their recommendations.

If applicable, is the statistical analysis and its interpretation appropriate?

I cannot comment. A qualified statistician is required.

Is the study design appropriate and is the work technically sound?

Yes

Is the work clearly and accurately presented and does it engage with the current literature?

Partly

Are the conclusions drawn adequately supported by the results?

Partly

Are sufficient details of methods and analysis provided to allow replication by others?

Partly

Are all the source data and materials underlying the results available?

No

Reviewer Expertise:

Government Data Processing, Automated Decision Making Systems, Data Transfers, PNR Processing and Transfers

I confirm that I have read this submission and believe that I have an appropriate level of expertise to confirm that it is of an acceptable scientific standard, however I have significant reservations, as outlined above.

## References

[1] : Lines of sight: on the visualization of unknown futures. *Citizenship Studies* .2009;13(1) : 10.1080/13621020802586628 17-30 10.1080/13621020802586628

[2] : Ligue des droits humains and the validity of the PNR Directive: Balancing individual rights and State powers in times of new technologies. *Common Market Law Review* .2023;60(Issue 3) : 10.54648/COLA2023054 839-862 10.54648/COLA2023054

[3] : Evaluatie PNR-Wet. *WODC rapport 3181* .2021; Reference source

[4] Extraterritorial Immigration Control.2010; 10.1163/ej.9789004172333.i-441 10.1163/ej.9789004172333.i-441

[5] : Beyond data protection concerns – the European passenger name record system. *Law, Innovation and Technology* .2021;13(2) : 10.1080/17579961.2021.1977221 398-421 10.1080/17579961.2021.1977221

[6] : CJEU PNR Decision Unplugs the ‘Black Box’. *European Data Protection Law Review* .2022;8(3) : 10.21552/edpl/2022/3/15 431-435 10.21552/edpl/2022/3/15

# Response

**Published:** 2025-04-28

**Authors:** Christiana Aposkiti

Thank you for your feedback. 

1. The relevant section has been adjusted. The main scope of the article is to present the findings from the survey. 

1. The relevant section has been adjusted. Every document we reviewed is added as a source to the document. 

1. The main purpose of the article is to present the findings of the study conducted with the aim of identifying the challenges of the PIUs. The recommendations are additional information that occurred from the survey results and are based on PIUs needs, being the result participants. Thus, the article evolves around the PIUs point of view and needs and does not move to a research about the legal implications the implementation of these recommendations would have as it has been considered a topic for future research based on the findings presented in the current article. This is also clarified in the section ‘Conclusion’ (i.e.g, ‘The identification of key challenges confronting PIUs serves as an initial step in this trajectory, paving the way for informed enhancements and ensuring the continued efficacy of travel intelligence initiatives in full respect of fundamental rights’). 

# Reviewer report: Reviewer response for version 1

**DOI:** 10.21956/openreseurope.19492.r42907

**Published:** 2024-08-29

**Authors:** Laura Drechsler, Abdullah Elbi

**Affiliations:** 1 Law, KU Leuven, Leuven, Flanders, Belgium

**1. Summary**

 The article “Navigating the challenges of passenger name record data and the way forward” by Christiana Aposkiti and Freideriki Makri analyses the operational challenges faced by Passenger Information Units (PIUs) within the EU in implementing the PNR Directive (EU) 2016/681. Through a survey conducted among PIUs from within and outside the EU, the study identifies two primary obstacles: the lack of standardized practices among stakeholders and the poor quality of PNR data. The authors note that these issues hinder the effective use of PNR data to ensure some of the security objectives of the EU, such as counter-terrorism and crime prevention. The authors conclude by proposing several recommendations, such as mandatory collection of specific data elements, and the automation of data quality controls to enhance the capabilities of PIUs. As the authors point out themselves, the study is limited by the unavailability of open data and the absence of a formal bias assessment. The conclusions drawn by the authors are generally supported by the survey results and relevant studies conducted by European Commission and academics, particularly in identifying the key challenges related to data quality and standardization.

 Overall, the article provides valuable insights into the challenges faced by PIUs in managing PNR data, and the recommendations offered have the potential to inform future policy and practice. It is generally well-structured and engages with relevant literature in the field of travel intelligence and PNR data usage.  Yet, the analysis of the legal aspects of PNR data use should be further refined as the current framing of relevant legal provisions as mere hindrances for PIUs misses their function as key guardrails which support the use of PNR technology in a responsible way to ensure the security objectives of the EU while respecting the fundamental rights of individuals. The following sub-sections will provide concrete recommendations with regard to the legal aspects (section II) and the methodology (section III) together with some general remarks (section IV).

**2. Legal aspects**

 The main issue for the discussion of the legal aspects is the superficiality in which the CJEU case C-817/19 is discussed. This case was the most relevant legal development for PNR data use in the EU in the last years, and it is much more nuanced that appears from the text of the article. This lack of nuance is especially reflected in the conclusion of the authors that cast all regulation as a barrier without distinction or much explanation (e.g. p. 1 or p. 6). The regulation of PNR data use can be criticized but the arguments of the authors would be stronger if they were further substantiated than the labelling as a barrier.

 Some of the insights of Case C-817/19 seem particularly pertinent for the discussion of the paper: (1) Due to the impact of PNR data use on individuals that are not suspected of any crime, the CJEU requires "strict" necessity and proportionality for the retention of PNR data, which is for example reflected in the interpretation of the retention periods noted by the authors. The reasoning is based on the need to balance the fundamental rights of individuals, such as privacy and personal data protection, with public interests. This balance could be more discussed and reflected on by the authors as it is key to the whole regulation of PNR in the EU. (2) For the CJEU a key factor in assessing the balance is to distinguish between applying the PNR Directive to intra-EU versus extra-EU flights. Member States must justify the application of PNR data collection to intra-EU flights with clear evidence that such measures are necessary and proportionate. This is a point that would be interesting if the authors could reflect upon. (3)  The judgment in Case C-817/19 also makes important findings on the use of automated or AI tools in the context of PNR data, e.g. by finding a need to have effective human intervention, ensure to a certain extent transparency of the systems used and requirements on data quality. This could be further reflected in the text as it links to some of the arguments the authors are making.

 A further interesting development to study is the opinion of the European Data protection Supervisor (EDPS) on the draft agreement for PNR data exchanges with Canada, which highlights some key EU legal requirements for PNR data use (see EDPS, ‘Opinion 15/2024 on the signing and conclusion of an Agreement between the EU and Canada on the transfer of Passenger Name Record (PNR) data’ (29 April 2024).

 Finally the references on the legal aspects of PNR data could be expanded. We would consider in particular following sources as potential useful material.:

- EDPS Opinions on recent PNR agreements (next to the one for Canada noted above):
  - Norway- https://www.edps.europa.eu/system/files/2023-10/2023-0903_opinion_en.pdf
  - Iceland - https://www.edps.europa.eu/system/files/2023-02/2022-1325_d0385_opinion_e-signed_en.pdf
  - Switzerland- https://www.edps.europa.eu/system/files/2023-10/2023-0920_opinion_en.pdf
- Vogiatzoglou, P., et al., 2020, [Ref-1]
- Thönnes, Christian; Vavoula, Niovi,2023 [Ref-2].
- Gerards, Janneke, 2023 [Ref-3].

**3. Methodological aspects**

 The study design, which involves a survey of PIUs, is appropriate for gathering insights into the operational challenges they face. However, due to the lack of detailed descriptions of the survey methodology, such as the number of questions asked, or the criteria used to analyze the responses, the outcome is not verifiable or very clear for readers. Including these details would significantly improve the transparency and replicability of the study. From a readers perspective, it would also be very helpful to see some outcomes of the survey partially as charts or graphs (or in another form of visualization) after anonymization respecting the security objectives of the project partners. Additionally, as also noted by authors the absence of a formal risk of bias assessment might weaken the robustness of the findings.

 The paper occasionally makes findings where it is not clear on what they are based (e.g. result of the survey, theme identified from the literature…). For example on top of p. 6 the authors note ‘The absence of automated technological solutions to monitor the validity and quality of the data received causes a huge workload for the PIU personnel’ but it is not clear if this is something the authors concluded from the survey or existing literature. Furthermore, considering the whole PNR discussion, e.g. in Case C-817/19 by the CJEU, it seems that to a certain extent PIUs are using automated solutions. It is therefore not clear whether the authors want to argue that there are no such tools available or that existing tools are not sufficient or reliable enough for this specific task. This could benefit from further elaboration.

 Finally, there is some uncertainty about the anonymity of the data. The authors note that ‘The participation to the survey was anonymous in order to protect the privacy and personal data of the respondent’ (p. 3) but they also acknowledge the collection of written informed consents at the end of the study *with ‘*All participants provided written informed consent by signing an information sheet and consent form for participation in the survey conducted as part of the TENACITy project’ (p. 8). It would be beneficial if the authors could clarify whether this latter consent was consent from a research ethics perspective or consent for the purposes of data protection. If it was consent from a data protection perspective, the authors should clarify whether the data was then perhaps pseudonymous instead of anonymous. This will also help potential readers to understand the methodology of the survey better.

 There are also some small formal issues that could benefit from addressing, such as a couple of typos. Furthermore, it would be great for the readability of the text if the authors could make sure that all acronyms are defined at the first use, e.g. on p. 4 the acronyms of IATA and ICAO.

**4. Other comments**

 The Introduction starts by introducing 9/11 as a cause for legislative efforts on PNR data in the US. This paragraph is not linked well to the rest of the introduction and the focus of the paper which is PNR data use in an EU context. It could be beneficial to the flow of the text to clearer link this first paragraph of the text to the rest, for example by discussing the effects of the US changes on the European legislative landscape or political efforts. It could also help to link to concrete EU developments or events (e.g., migration influx or terrorist attacks etc.). For example, the API Directive was also a response to the 2004 Madrid terrorist attack. The introduction further mentions EES and ETIAS as border management tools but misses to distinguish them from the use of PNR/API, which makes it difficult to understand their role in the discussions of the paper. Finally, it might be worth further expanding on the background of the PNR Directive itself, which had not had the easiest legislative journey with several earlier attempts to legislate in that area failing (e.g. in 2007).

 In the recommendation section, the first and second recommendation are rather similar. It would be beneficial to the reader to either further clarify how they differ or combine them into one longer recommendation. The third recommendation (‘A recommendation stemming from…’) is formulated in a very passive manner and might be more powerful if rephrased. The discussion of this recommendation would also benefit from a (brief) investigation of the impact of the AI Act with its potential rules on the suggested mechanisms for real-time feedback and validation.

If applicable, is the statistical analysis and its interpretation appropriate?

Not applicable

Is the study design appropriate and is the work technically sound?

Yes

Is the work clearly and accurately presented and does it engage with the current literature?

Yes

Are the conclusions drawn adequately supported by the results?

Yes

Are sufficient details of methods and analysis provided to allow replication by others?

Partly

Are all the source data and materials underlying the results available?

No

Reviewer Expertise:

Data protection law, border management law, fundamental rights, EU law

We confirm that we have read this submission and believe that we have an appropriate level of expertise to confirm that it is of an acceptable scientific standard, however we have significant reservations, as outlined above.

## References

[1] : From Theory To Practice: Exercising The Right Of Access Under The Law Enforcement And PNR Directives. *JIPITEC* .2020; Reference source

[2] : Automated predictive threat detection after Ligue des Droits Humains. *Verfassungsblog: On Matters Constitutional* .2023; 10.17176/20230508-204551-0 10.17176/20230508-204551-0 Reference source

[3] : Machine learning and profiling in the PNR system. *VerfBlog* .2023; 10.17176/20230508-204540-0 10.17176/20230508-204540-0

# Response

**Published:** 2025-04-28

**Authors:** Christiana Aposkiti

Thank you for your feedback.   Legal aspects  

- We recognize the importance of providing a more nuanced discussion of Case C-817/19 and its implications for the regulation and use of PNR data in the EU. In response to your comments, we have revised the section on legal matters to address your comments.  Additionally, we have incorporated references to recent EDPS opinions and relevant legal scholarship to strengthen the discussion and provide further context.  

- Language has been adjusted in the relevant sections 

- We have expanded the legal aspects section to include a more detailed discussion of the key points raised by the judgment. While these additions aim to provide a more comprehensive reflection on the legal aspects, we note that a detailed legal analysis of Case C-817/19 is outside the primary scope of this study, which focuses on the operational challenges faced by PIUs. 

Methodological aspects 

- The number of questions is added in the section methodology.  

- Graphs were added and can be found in the extended data section. 

- The absence of a formal risk or bias assessment is mentioned as one of the key limitations of the research. 

- When a finding is based on existing literature instead of the survey, the reference is cited within the text. If a finding is not followed by a citation then it occurred solely from the survey. 

- The ‘Consent to participate’ section has been adjusted accordingly. 

Other comments  Relevant sections have been adjusted.
